# Grifolin, neogrifolin and confluentin from the terricolous polypore *Albatrellus flettii* suppress KRAS expression in human colon cancer cells

**DOI:** 10.1371/journal.pone.0231948

**Published:** 2020-05-05

**Authors:** Almas Yaqoob, Wai Ming Li, Victor Liu, Chuyi Wang, Sebastian Mackedenski, Linda E. Tackaberry, Hugues B. Massicotte, Keith N. Egger, Kerry Reimer, Chow H. Lee

**Affiliations:** 1 Chemistry and Biochemistry Program, University of Northern British Columbia, Prince George, British Columbia, Canada; 2 Ecosystem Science and Management Program, University of Northern British Columbia, Prince George, British Columbia, Canada; Marshall University, UNITED STATES

## Abstract

In our search for bioactive mushrooms native to British Columbia, we determined that the ethanol extracts from fruiting bodies of the terrestrial polypore *Albatrellus flettii* had potent anti-cell viability activity. Using bioassay-guided fractionation, mass spectrometry and nuclear magnetic resonance, we successfully isolated three known compounds (grifolin, neogrifolin and confluentin). These compounds represent the major anti-cell viability components from the ethanol extracts of *A*. *flettii*. We also identified a novel biological activity for these compounds, specifically in down-regulating KRAS expression in two human colon cancer cell lines. Relatively little is known about the anti-cell viability activity and mechanism of action of confluentin. For the first time, we show the ability of confluentin to induce apoptosis and arrest the cell cycle at the G2/M phase in SW480 human colon cancer cells. The oncogenic insulin-like growth factor 2 mRNA-binding protein 1 (IMP1) has been previously shown to regulate KRAS mRNA expression in colon cancer cells, possibly through its ability to bind to the KRAS transcript. Using a fluorescence polarization assay, we show that confluentin dose-dependently inhibits the physical interaction between KRAS RNA and full-length IMP1. The inhibition also occurs with truncated IMP1 containing the KH1 to KH4 domain (KH1to4 IMP1), but not with the di-domain KH3 and KH4 (KH3&4 IMP1). In addition, unlike the control antibiotic neomycin, grifolin, neogrifolin and confluentin do not bind to KRAS RNA. These results suggest that confluentin inhibits IMP1-KRAS RNA interaction by binding to the KH1&2 di-domains of IMP1. Since the molecular interaction between IMP1 and its target RNAs is a pre-requisite for the oncogenic function of IMP1, confluentin should be further explored as a potential inhibitor of IMP1 *in vivo*.

## Introduction

There have been relatively few studies exploring the medicinal potential of compounds from wild mushrooms native to British Columbia (BC), Canada. Therefore, we have begun to study medicinal properties of selected native fungi, focusing on three bio-assays relevant to cancer: anti-cell viability, immuno-stimulatory and anti-inflammatory activities [1,2]. Screening over one hundred mushroom species occurring in north-central BC, we have documented that approximately 25% possess potent anti-cell viability activity. Among the mushrooms that exhibited some of the most potent anti-cell viability activity was *Albatrellus flettii* and, specifically, its ethanol extracts.

*Albatrellus flettii* (family Albatrellaceae) is an attractive terricolous polypore, often with a distinct blue-gray colouring on the cap; it is commonly found in western North America ranging from Alaska to California and Mexico, including throughout BC, Alberta, Washington, Oregon, Idaho and Wyoming [3]. Although there are several studies on the phytochemistry of the genus *Albatrellus* [4–9], only two reported on *A*. *flettii* [10,11]. The first focused predominantly on the isolation of meroterpenoid pigments responsible for the blue color of *A*. *flettii* [10]. In the second study, three compounds (grifolin, neogrifolin and confluentin) were isolated from *A*. *flettii* (collected from Oregon), and these were documented to have anti-bacterial activities against *Bacillus cereus* and *Enterococcus faecalis* [11]. Spectroscopic data were not provided and there was no evidence of purity of compounds. To date, *A*. *flettii* has not been systematically studied for its anti-cell viability activity against human cancer cells nor have compounds been isolated with such activity.

KRAS is a frequently mutated oncogene in human cancer cells with the highest prevalence in pancreatic adenocarcinomas (90%), colorectal cancers (45%) and lung cancers (35%) [12]. The KRAS gene encodes a small membrane protein which is active when bound to GTP and consequently activates a number of downstream biochemical pathways leading to cell growth, differentiation and survival. Mutated KRAS is locked onto the GTP-bound “on” state and continuously activates the downstream biochemical effectors that can lead to tumorigenesis [13,14]. Recent developments in finding RAS inhibitors include: (i) a bicyclic diterpenoid lactone which blocks GDP-GTP exchange and inhibits both the wild-type and oncogenic KRAS signaling by binding to the switch regions of RAS [15], and (ii) inhibitors which irreversibly target a type of KRAS mutant by forming a covalent attachment [16,17]. Recent studies using preclinical mouse models have shown regression of KRAS-driven tumors using highly potent and specific siRNA [18–20] and antisense oligonucleotide [21] targeting KRAS expression. This suggests that inhibition of KRAS expression is an effective approach in anti-KRAS therapy.

In the course of our search for anti-cell viability metabolites, we isolated three known major compounds (grifolin, neogrifolin and confluentin) from the ethanol extracts of fruiting bodies of *A*. *flettii* using a bioassay-guided fractionation approach. We report here, for the first time, that grifolin, neogrifolin and confluentin suppressed KRAS expression in human colon cancer cells. Confluentin also inhibited the physical association between KRAS RNA and insulin-like growth factor 2 mRNA-binding protein 1 (IMP1). This suggests its further potential to inhibit these oncogenic biomolecules in cell lines and animal models.

## Materials and methods

### General experimental procedures

1D and 2D NMR spectra were recorded on a Bruker 300 MHz NMR spectrometer with a 5 mm Kimble NMR tube (Rockwood, TN, USA). All HPLC analyses were performed on Agilent 1260 Infinity Systems with UV detector, and mass spectrometry were done using Agilent 6120 Single Quad MS. For analytical purposes, a reverse phase column Phenomenex Aqua^®^ C18 (250 x 4.6 mm, 5 μm) was used. Solvent A: water, solvent B: acetonitrile, gradient system: 90% B increasing by 10% every 5 min for 15 min maintaining at 90% B for 20 min, flow rate = 1.0 mL min^-1^, UV detection at 279 nm. For semi-preparative purification, an Agilent Zorbax Eclipse XBD-C18 (9.4 x 250 mm, 5 μm) was used. Solvent A was water and solvent B was acetonitrile. The following conditions were used: gradient system, 90% B for 20 min; flow rate = 2.0 mL min^-1^; UV detection at 279 nm. HPLC grade solvents were purchased from BDH (Mississauga, ON, Canada). Eagle’s minimum essential medium (EMEM) and phosphate-buffered saline (PBS) were obtained from Lonza (Walkersville, MO, USA). Fetal bovine serum (FBS) was obtained from Life Technologies Inc. (Grand Island, NY, USA). KRAS mouse monoclonal antibodies (1:500 dilution) were from EMD Millipore (MABS194 clone 234–4.2; Etobicoke, ON, Canada) and Abnova (H00003845-M01 clone 3B10-2F2; Taoyuan City, Taiwan), and the mouse monoclonal GAPDH antibody (G8795; clone GAPDH-71.1) (1:10,000 dilution) was from Sigma-Aldrich (St. Louis, MO, USA). Cleaved Caspase-3 antibody (Asp175; #9661) (1:1000 dilution) was from Cell Signaling Technology Inc. (MA, USA).

### Fungal collection and identification

Fruiting bodies of *A*. *flettii* were collected near the Twin Falls Recreation Site, Smithers, BC, Canada, on September 3, 2016 (CL128) and September 20, 2017 (CL151). Voucher specimens for these collections were deposited at the University of Northern British Columbia, Canada. The identity of *A*. *flettii* was confirmed using previously described genetic protocols [1,2,22]. The DNA sequence of collections CL128 and CL151 were identical and had highest sequence similarity to a collection of *A*. *flettii* GenBank JF899544.1 (99.82% identity/100% coverage).

### Extraction and isolation

*Albatrellus flettii* specimens were sequentially extracted with 80% ethanol, 50% methanol, water and 5% NaOH as previously described [1,2]. Solvents were completely removed by rotovap and powdered extracts were obtained by lyophilization. The 5% NaOH extract (E4) was pH neutralized and dialyzed in water before subjected to lyophilization. The 80% ethanol (E1) and 50% methanol (E2) extracts were resuspended in methanol while the water (E3) and 5% NaOH (E4) extracts were resuspended in water.

For the isolation of compounds, oven-dried and powdered *A*. *flettii* fruiting bodies (70 g) were extracted with 80% ethanol at room temperature and filtered. The filtrate was evaporated and lyophilized to obtain a crude EtOH extract (7.1 g) that was resuspended in CHCl_3_ and then partitioned with water (1:1). The lower layer containing CHCl_3_ (3.2 g of solid material recovered) had anti-cell viability activity; it was subjected to five runs (640 mg/run at 80 mg/mL) of Sephadex LH-20 column chromatography (1000 x 26 mm i.d., in methanol) to yield 50 fractions (10 mL per fraction). Fractions 30–36, which exhibited anti-cell viability activity, were first subjected to an analytical HPLC reverse phase column Phenomenex Aqua^®^ C18. Based on the UV spectra overlay, compounds eluting at 9.7 min, 13.5 min and 23.5 min were the most abundant (S1 Fig) and exhibited anti-cell viability activity. The remaining fractions 30–36 from Sephadex LH-20 column were subjected to semi-preparative HPLC purification using an Agilent Zorbax Eclipse XBD-C18 column. The compound obtained at the retention t_R_ = 9.5 min was later confirmed by MS (S2 Fig) and NMR (S3–S6 Figs) to be grifolin. The compound obtained at the retention t_R_ = 13.5 min was later confirmed by MS (S7 Fig) and NMR (S8–S11 Figs) to be neogrifolin. The compound obtained at the retention t_R_ = 23.5 min was later confirmed by MS (S12 Fig) and NMR (S13–S18 Figs) to be confluentin. Grifolin, neogrifolin and confluentin were dissolved in dimethyl sulfoxide (DMSO) to make a 50 mM stock solution for biological assays.

### Cytotoxicity assay

The following cell lines purchased from American Type Culture Collection were used: HeLa (cervix epithelioid carcinoma; CCL-2) and SW480 (colon adenocarcinoma; CCL-228). The cell line HT29 (colon adenocarcinoma) was obtained from Dr. Ranjana Bird (UNBC, 2014). The cytotoxic MTT assay was used as previously described [1,2,22]. Briefly, cells were plated at a density of 1.5 x 10^4^ cells/well in 96-well plates. After 24 hours, cells were treated with various concentrations (1, 0.75, 0.5, 0.25, and 0.1 mg/mL) of crude extracts E1, E2, E3 and E4 for 48 hours. Water was used as control for E3 and E4 while methanol (5%) was used as control for E1 and E2. Absorbance data were expressed relative to the respective controls, water or 5% methanol, taken as 100% cell viability. For studies with compounds, cells were treated with various concentrations (80, 60, 40, 20, 10, 5, 2.5, 1.25, and 0.625 μM) of grifolin, neogrifolin and confluentin or with 0.1% DMSO for 48 hours. All absorbance data were expressed relative to the control, 0.1% DMSO, taken as 100% cell viability. We have previously used 25 μM cis-platinum, a known inducer of apoptosis, as a positive control and showed that 95% anti-cell viability was achieved.

### Western blot analysis

Cells were seeded in 6-well plates at a density of 2.5 x 10^5^ cells/well and treated with grifolin, neogrifolin and confluentin or with 2% DMSO for 24 and 48 hours. Cell lysates were prepared as previously described [23]. Protein samples were resolved on a 10% SDS-PAGE and transferred onto a nitrocellulose membrane. BLUelf prestained protein ladder (FroggaBio) was used. Anti-mouse IgG-HRP (1:4,000, Promega) was used as secondary antibody. All blots were visualized using chemiluminescent detection system with the FluorChem Q Imager (ProteinSimple, CA, USA) and analyzed using the AlphaView Q software (ProteinSimple).

### Fluorescence polarization analysis

A fluorescent polarization method to study the physical interaction between IMP1 and KRAS RNA is reported here for the first time. The method employs a recombinant IMP-1 protein and fluorescent-labeled 44-nt KRAS RNA. The preparation of recombinant full-length wild-type IMP1 protein and truncated IMP1 protein KH34 (containing only KH3 and KH4 domains) has been previously described [23]. The following were mixed in a Thermo Scientific micro-Fluor 384-well microplate: 8 μL protein, 3 μL Thermo Scientific 44-nt fluorescein labeled KRAS Probe (5’AUGGAGAAACCUGUCUCUUGGAUAUUCUCGACACAGCAGGUCAU-6-FL-3’) and 4 μL binding buffer (50 mM Tris-Cl pH 7.4, 12.5 mM EDTA pH 8, 25% glycerol, 0.01% Triton-X and H_2_O). The 39-nt fluorescein labeled CD44 probe was 5’AAAUUAGGGCCCAAU UAAUAAUCAGCAAGAAUUUGAUCG-6-FL-3’. The final concentration of IMP1 and KRAS probe was 300 nM and 10 nM, respectively. To each well, 4 μL of grifolin, neogrifolin, confluentin or 5% DMSO was added for a final concentration of 5, 10, 20, 40, 80 or 100 μM. Plates were incubated at 37°C for 30 min prior to reading at 485/20 nm excitation and 528/20 nm emission using the Bio-Tek Synergy 2 plate reader.

### Flow cytometry apoptosis and cell cycle analyses

Cells were plated at 2.5 x 10^5^ cells/well in 6-well plates and treated with grifolin, confluentin or 2% DMSO for 24 hours. Untreated cells were included as control. Cells were trypsinized followed by centrifugation and washed twice with phosphate-buffered saline. Live cells were stained with PE Annexin V and 7-AAD according to the manufacturer’s instructions for Apoptosis Detection Kit I (BD Pharmingen) and analyzed by flow cytometry using a BD FACSMelody cell sorter (BD Biosciences) and BD FACSChorus software (V 1.0). For cell cycle analyses, BD cycletest plus DNA reagent kit was used.

### Electrophoretic mobility shift assay

Human KRAS (accession # NM_033360) cDNA purchased from OriGene Technologies Inc. was used as template for the generation of PCR-amplified DNA fragments. The primer set used for amplifying KRAS cDNA nts 1–185 were: forward, 5’-GGATCCTAATACGACTCACTATAGGATGACTGAATATAAACTT-3’ and reverse, 5’-TCATGACCTGCTGTGTCG-3’. The primer sets for amplifying 5’end and 3’end truncated KRAS cDNAs corresponding to nts 1–185 has been described previously [24]. One μg of PCR-amplified DNA templates was used directly for *in-vitro* transcription by T7 RNA polymerase as previously described [23–25]. The purified, radiolabeled RNA was phenol/chloroform extracted followed by ethanol precipitation. Specific activity of the RNA was then determined by scintillation counting. The electrophoretic mobility shift assay (EMSA) was conducted in essentially the same manner as previously described [23–26]. Radiolabeled RNAs (20,000 cpm) were used and IMP1 concentrations varied from 0 to 540 nM. Free and bound RNAs were resolved in 8% non-denaturing polyacrylamide gel and visualized using Cyclone Storage Phosphor Imager System (Packard Instrument). Images were analyzed and quantified using Optiquant software version 4.0.

### Statistical analysis

The cytotoxic MTT assays were performed in triplicate (3 wells/treatment) and the absorbance reading from the respective control was taken as 100% cell viability. Two additional experiments were conducted and the mean IC_50_ values from three independent experiments (n = 3) were obtained. For Western blot analysis, the intensity of KRAS and Cleaved Caspase-3 bands were normalized to their respective GAPDH bands and then expressed relative to the control DMSO (taken as 1.0). After additional biological replicates were conducted, one-way ANOVA or Student’s t-test was performed to compare the drug-treated samples with DMSO-treated samples (n = 3 or 5). Similarly for fluorescence polarization and flow cytometry experiments, three independent experiments (n = 3) were performed. Data were analyzed using GraphPad Prism version 8.0.2 (La Jolla, CA). A p-value < 0.05 was considered as statistically significant.

## Results and discussion

### Isolation and identification of grifolin, neogrifolin and confluentin

**Fig 1:**
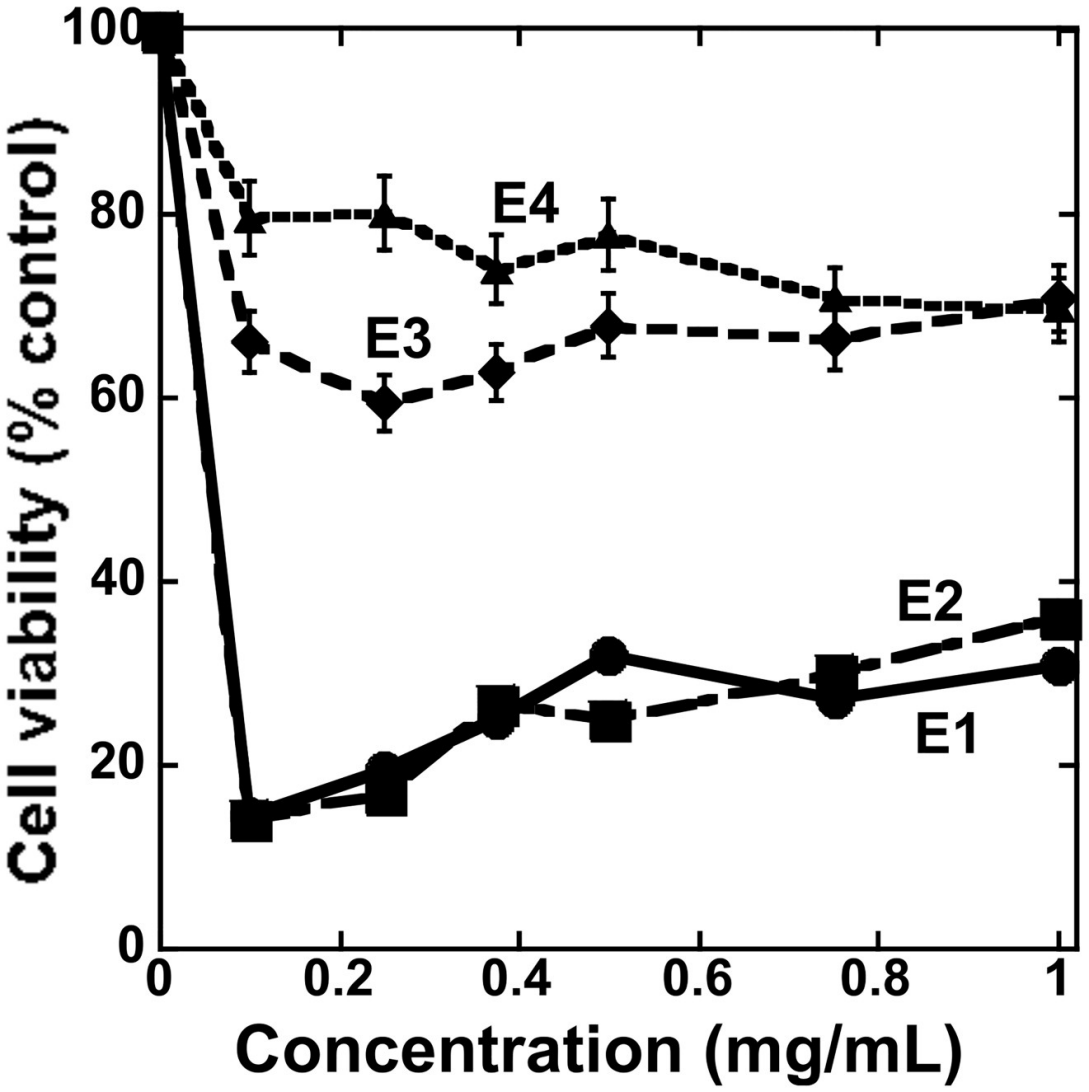
Dose-dependent effect of crude extracts from *A*. *flettii* on the viability of HeLa human cervical cancer cells. Cells were treated for 48 hours with various concentrations of E1 (80% ethanol), E2 (50% methanol), E3 (water) and E4 (5% NaOH) extracts from *A*. *flettii*. The concentrations of extracts used were: 1, 0.75, 0.5, 0.25 and 0.1 mg/mL. Anti-cell viability was measured by MTT assay. Results shown represent two biological replicates. Error bars are standard deviation (S.D.).

**Fig 2:**
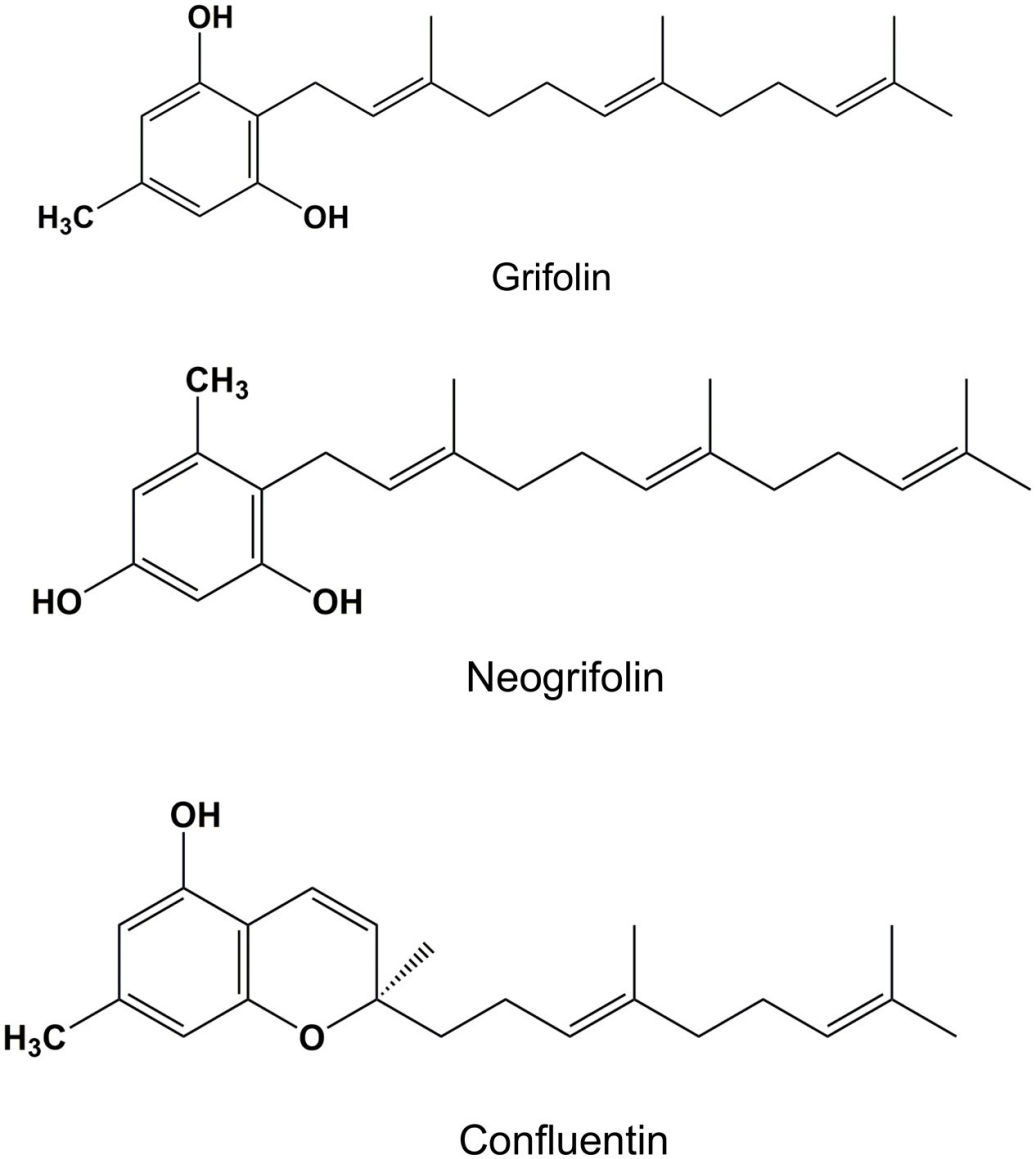
Chemical structure of grifolin, neogrifolin and confluentin isolated from *A*. *flettii*.

*Albatrellus flettii* specimens were sequentially extracted with 80% ethanol, 50% methanol, water and 5% NaOH. The water (E3) and 5% NaOH (E4) extracts displayed weak anti-cell viability activity while the 80% ethanol (E1) and 50% methanol (E2) extracts showed potent anti-cell viability activity against HeLa human cervical cancer cells (Fig 1). At and above 0.4 mg/mL, both E1 and E2 showed increase in cell viability and this is due to the fact that they contain constituents that react with the medium or MTT to produce colored solutions—thereby producing false positive results. Similar effects have been observed for a number of fungal extracts [2]. Consequently, we focused the study on the isolation of anti-cell viability compounds from the 80% ethanol extract. Chloroform:H_2_O (1:1) extraction, Sephadex LH-20 column chromatography, HPLC-MS (S2, S7 and S12 Figs) and NMR (S3–S6, S8–S11 and S13–S18 Figs) analyses of the ethanol extracts led to the isolation and identification of grifolin, neogrifolin and confluentin (Fig 2) as the three major anti-cell viability compounds. Based on HPLC UV spectra (S1 Fig), the abundance of grifolin, neogrifolin and confluentin was estimated to be 32.6%, 52.0% and 8.1%, respectively. A previous study cited anti-bacterial activity for grifolin, neogrifolin and confluentin isolated from *A*. *flettii*, however, abundance values were not reported [11].

### Grifolin, neogrifolin and confluentin are cytotoxic to human colon cancer cells

**Fig 3:**
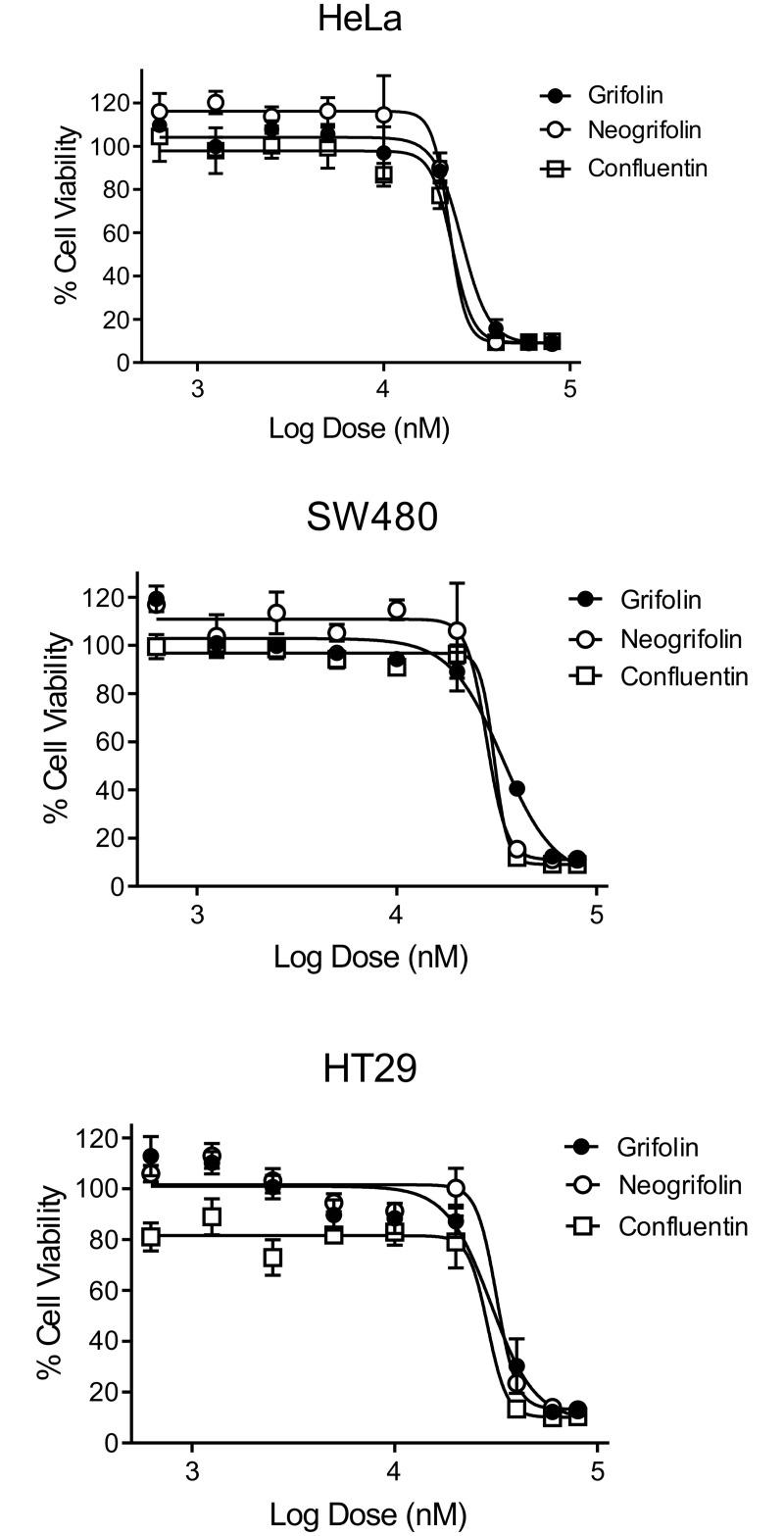
Grifolin, neogrifolin and confluentin inhibited cell viability in human cancer cells. HeLa human cervical cancer cells and human colon cancer cells (SW480 and HT29) were treated with various concentrations of grifolin, neogrifolin and confluentin for 48 hours. The concentrations of compounds used were: 80, 60, 40, 20, 10, 5, 2.5, 1.25 and 0.625 μM. Cell viability was determined using the cytotoxic MTT assay. Each dose was tested in triplicate in each experiment and the data shown is a representation of at least three independent experiments (n = 3).

We evaluated grifolin, neogrifolin and confluentin for anti-cell viability activity against HeLa cells and two human colon cancer cell lines SW480 and HT29. Grifolin has IC_50_ values of 27.4 ± 2.2 μM (n = 3), 35.4 ± 2.4 μM (n = 4) and 30.7 ± 1.0 μM (n = 3) against HeLa, SW480 and HT29 cells, respectively (Fig 3). This is consistent with a previous report of IC_50_ values of 34 μM and 27 μM for grifolin from *Albratrellus confluens* on HeLa and SW480 cells [27]. Neogrifolin has IC_50_ values of 24.3 ± 2.5 μM (n = 3), 34.6 ± 5.9 μM (n = 4) and 30.1 ± 4.0 μM (n = 4) against HeLa, SW480 and HT29 cells, respectively (Fig 3). Although there are no reports of anti-cell viability activity by neogrifolin on HeLa, SW480 and HT29 cells, it has been shown to have IC_50_ values of 25–50 μM against human osteosarcoma cells [28]. Confluentin has IC_50_ values of 25.9 ± 2.9 μM (n = 3), 33.5 ± 4.0 μM (n = 3) and 25.8 ± 4.1 μM (n = 3) against HeLa, SW480 and HT29 cells, respectively (Fig 3). Prior to the present study, confluentin had not been assessed on human colon cancer cells. Confluentin from *A*. *ovinus* has been reported to have IC_50_ values in the range of 15–23 μM on the following human cancer cell lines: HL-60, SMMC 7712, A-549 and MCF-7 [5]. In summary, grifolin, neogrifolin and confluentin isolated from *A*. *flettii* have anti-cell viability activity against HeLa human cervical cancer cells and two human colon cancer cell lines, SW480 and HT29. The IC_50_ values reported here are similar to those reported by others for these compounds derived from fungal species in the same genus, tested against either the same or different cancer cell lines. We have not assessed grifolin, neogrifolin and confluentin on normal cells immortalized in culture. It is highly likely that these compounds are also cytotoxic to immortalized normal cells.

### Confluentin induces apoptosis and arrests cell cycle in human colon cancer cells

**Fig 4:**
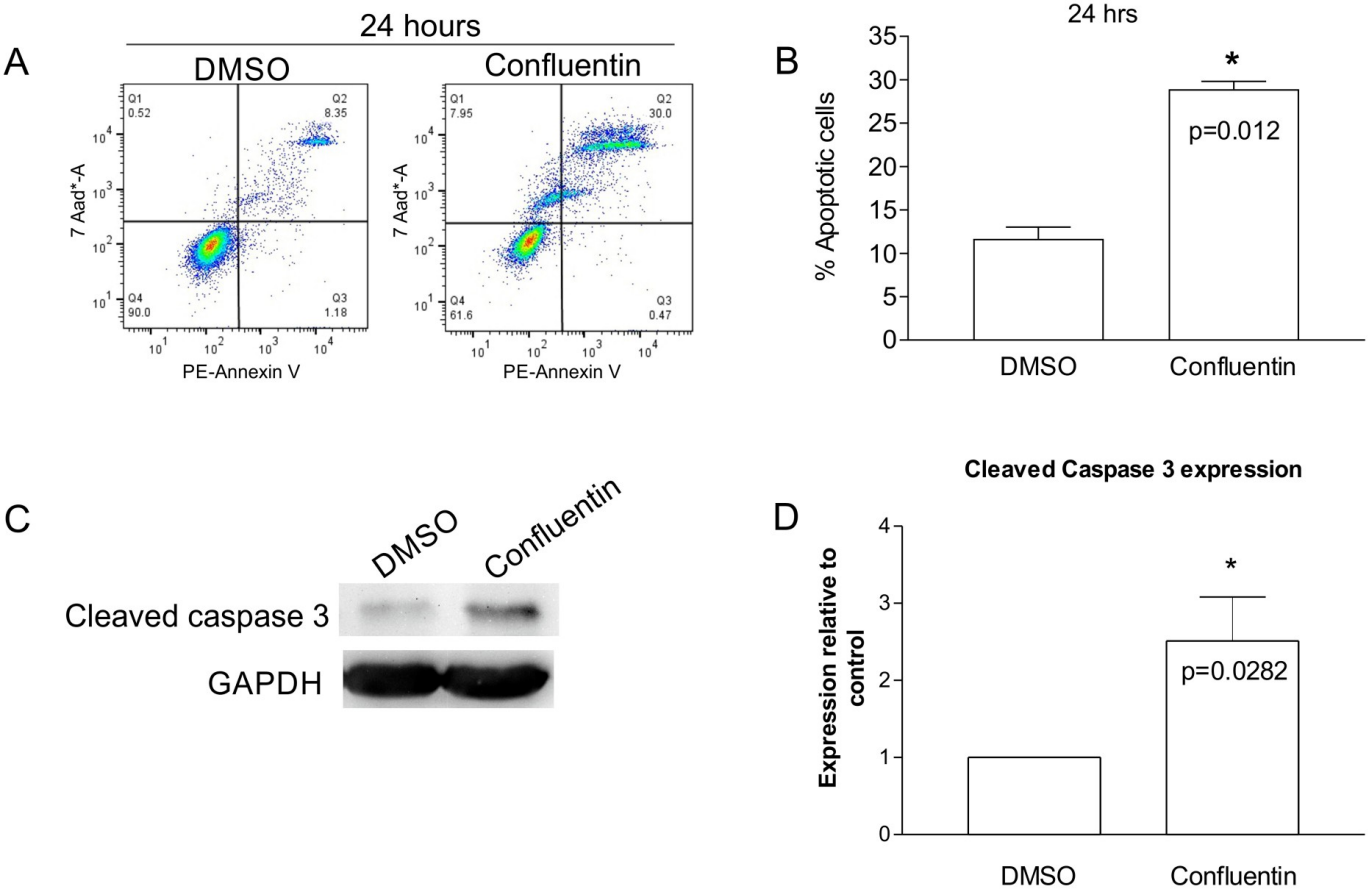
Effect of confluentin on apoptosis in SW480 human colon cancer cells. (A) SW480 cells were treated with 2% DMSO or 50 μM confluentin for 24 hours after which cells were analyzed by flow cytometry to assess for necrosis (Q1), late apoptosis (Q2), early apoptosis (Q3) and live cells (Q4). Data shown represents three independent experiments (n = 3). (B) The percentages of cells in late apoptosis (Q2) treated with 2% DMSO or 50 μM confluentin from three independent experiments were combined and shown to be statistically significant (p < 0.05). (C) As in (A), SW480 cells treated with 2% DMSO or 50 μM confluentin for 24 hours followed by isolation of cell lysates. Western blot analyses were performed and data shown is a representative from five independent experiments (n = 5). (D) The level of Cleaved Caspase-3 in confluentin-treated cells was normalized to that of GAPDH and expressed relative to DMSO-treated cells (taken as 1.0). The plotted graph is from five independent experiments. For statistical analysis, a Student’s t-test was performed (p < 0.05). Error bars are S.D.

Grifolin, mostly isolated from *A*. *confluens*, has been extensively studied for its anti-cell viability mechanism [27,29–34]. Similarly, neogrifolin, also isolated from *A*. *confluens*, has been shown to induce release of cytochrome c and to activate caspases 3 and 9 leading to apoptosis [28]. In contrast, no study exists on the anti-cell viability mechanism of confluentin. Here, we used flow cytometry and Western blot analyses to assess the effect of confluentin on cell apoptosis. After 24 hours of treatment with 50 μM confluentin, 30% of SW480 cells underwent apoptosis compared to 8.4% of cells treated with 2% DMSO (Fig 4A) and such an effect was confirmed with additional experiments (Fig 4B). We also found that 50 μM confluentin increased Cleaved Caspase-3 expression 2.5-fold compared to DMSO-treated cells (Fig 4C and 4D), further confirming the ability of confluentin to induce apoptosis.

**Fig 5:**
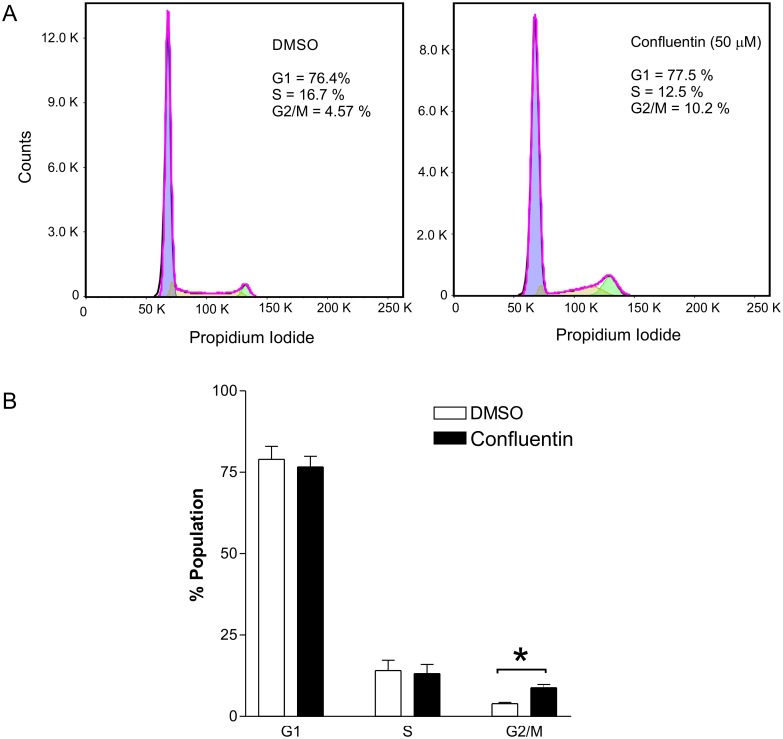
Effect of confluentin on cell cycle in SW480 human colon cancer cells. (A) SW480 cells treated with 2% DMSO or 50 μM confluentin were analyzed by flow cytometry for cell-cycle distribution. (B) The relative percentages of cells in different phases of cell cycle were combined from three independent experiments (n = 3) and plotted as shown. Two-way ANOVA was performed and asterisk indicates significant differences (p < 0.001). Error bars are S.D.

Using flow cytometry, we also assessed the effect of confluentin on the cell cycle in SW480 cells. We determined that approximately 10% of cells were arrested at G2/M phase following 24 hours treatment with 50 μM confluentin compared to 4.6% of cells when treated with the control 2% DMSO (Fig 5A). Two additional biological replicates confirmed that confluentin significantly arrests SW480 cells at the G2/M phase. Our study also showed that confluentin had no effect on cells at the G1 and S phase (Fig 5B).

In summary, we report here for the first time that confluentin induces apoptosis and arrests the cell cycle at the G2/M phase, both mechanisms that could contribute to anti-cell viability activity on SW480 cells (Fig 3).

### Grifolin, neogrifolin and confluentin suppress KRAS expression

**Fig 6:**
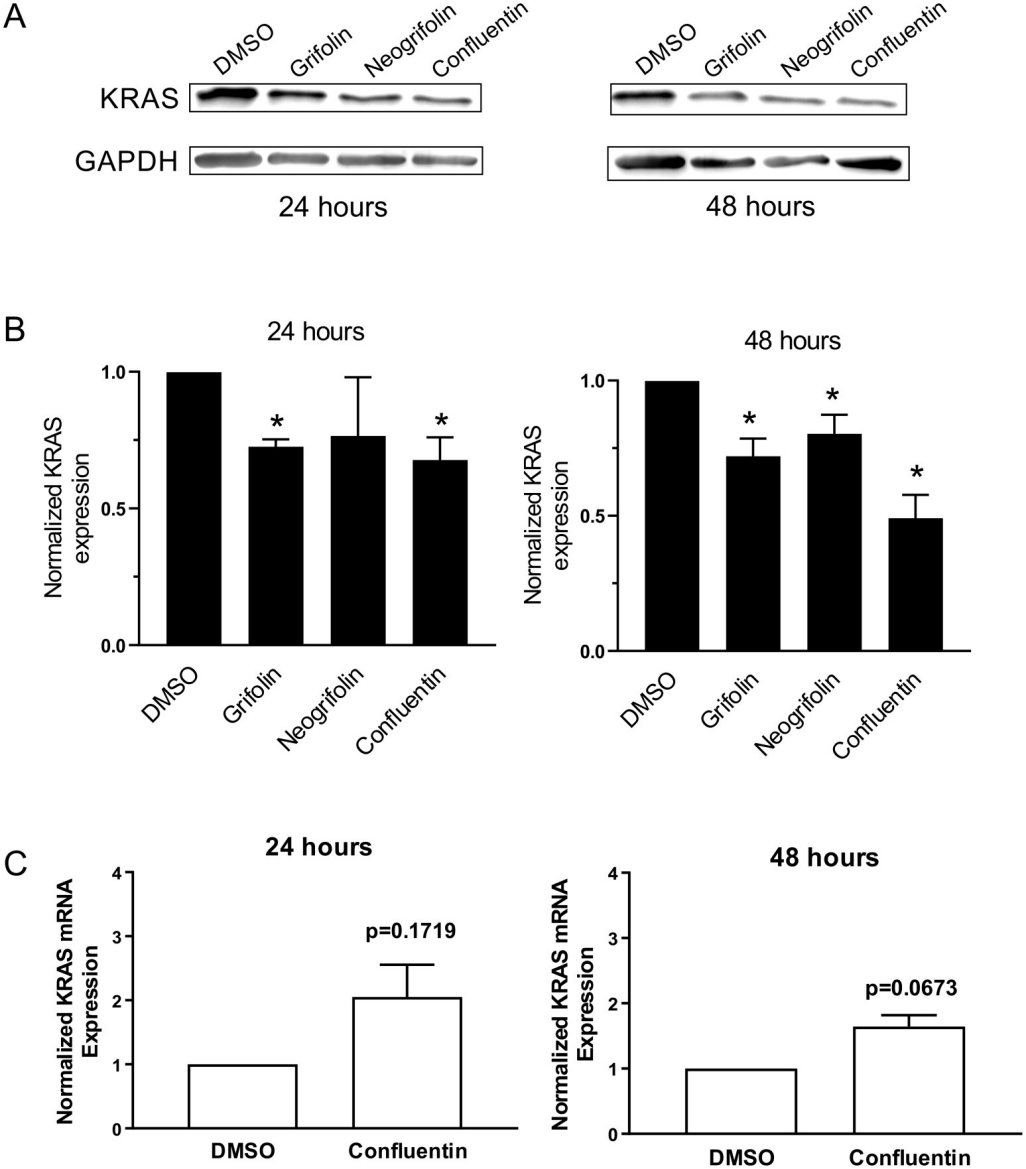
Effect of grifolin, neogrifolin and confluentin on KRAS expression in SW480 human colon cancer cells. SW480 cells were treated with 2% DMSO or 50 μM grifolin, neogrifolin and confluentin for 24 and 48 hours. (A) A representative immunoblot is shown. (B) KRAS band intensity was normalized to their respective GAPDH intensity and expressed relative to the control DMSO (taken as 1.0). Data shown are mean ± SD pooled from three independent experiments (n = 3). * = p < 0.01 versus DMSO. (C) Total RNA isolated from treated cells was subjected to quantitative real-time PCR. The level of KRAS mRNA from confluentin-treated cells was normalized to that of GAPDH mRNA and expressed relative to that of DMSO-treated cells. Data shown was pooled from three biological replicates (n = 3). A student’s t-test was performed and error bars are S.D.

KRAS is an elusive oncotarget and much effort continues to focus on targeting it by directly inhibiting its function, biogenesis and expression [14,35]. Furthermore, constitutively active KRAS has been linked to increased cell proliferation [36–38]. Therefore, we investigated whether grifolin, neogrifolin and confluentin have any effect on the expression of KRAS in human colon cancer cells. Upon treatment for 48 hours at 50 μM, all three compounds showed significant inhibition on KRAS expression in SW480 cells, which carry the G12V KRAS mutation (Fig 6). After 24 hours of treatment, only grifolin and confluentin had a significant effect. Although not statistically significant, neogrifolin also showed an inhibitory pattern at 24 hours (Fig 6). In addition, we assessed the three compounds on KRAS expression in the HT29 human colon cancer cell line, which carries the wild-type KRAS. At 20 and 50 μM, we found similar inhibition on KRAS expression by grifolin, neogrifolin and confluentin (S19 Fig). We also determined whether confluentin, the most effective amongst the three compounds, had any effect on KRAS mRNA level in SW480 cells. Our results showed that confluentin had no significant effect on KRAS mRNA levels at 24 and 48 hours after treatment (Fig 6C). Taken together, our findings suggest that grifolin, neogrifolin and confluentin decrease KRAS expression at the level of translation rather than at the level of mRNA degradation.

In summary, these results are the first to show the ability of grifolin, neogrifolin and confluentin to suppress KRAS expression in human colon cancer cells regardless of whether the cells carry KRAS mutation or the wild-type KRAS. Further experiments are clearly required to decipher the exact mechanism whereby these compounds suppress KRAS expression.

### Establishing a fluorescence polarization method to study IMP1-RNA interactions

**Fig 7:**
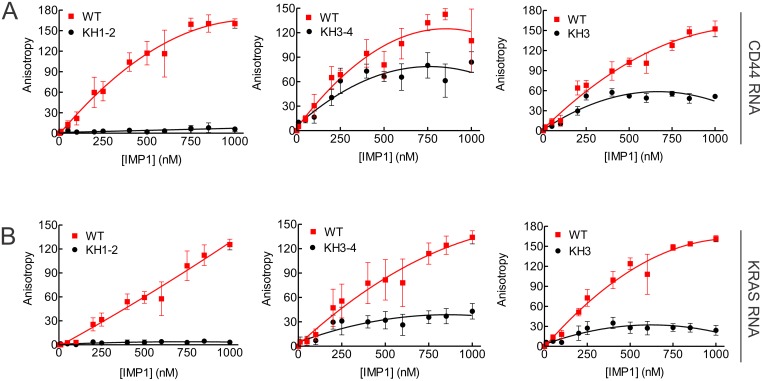
Establishing the fluorescence polarization method to study IMP1-RNA interaction. Fluorescence polarization analysis of the interaction between the wild-type IMP1 and its point-mutation variants (KH1-2, KH3-4 and KH3) with the fluorescein-labeled 39-nt CD44 (A) and the 44-nt KRAS RNAs (B). Results shown are representative of three separate experiments (n = 3).

One of the major criteria for successful use of fluorescence polarization (FP) to study nucleic acid-protein interaction is the requirement of a small molecular weight fluorescently-labeled substrate, which in this case is an RNA [39]. Using a gel shift assay, we mapped a number of RNA substrates to determine the smallest region still capable of binding to IMP1 [25,26], including CD44 [40] and KRAS RNA [24]. We previously showed that a 35-nt CD44 RNA spanning nts 2862–2896 is the minimal region capable of binding IMP1 [40]. Based on a report that IMP1 has high affinity for the coding region and 3’UTR of KRAS mRNA [38], we proceeded to map the smallest region(s) of KRAS mRNA that can bind IMP1. Our results show that nts 1–185 has one of the highest affinities for IMP1 with a Kd of 171 ± 18 nM [24]. In this study, nts 1–185 of KRAS mRNA was further mapped by truncation from the 5’end and 3’end and results are shown in S20 Fig. Gel shift analysis of seven truncated fragments show that nts 93–185 is the region of IMP1 binding. Further mapping of nts 93–185 finally revealed the 39-nt KRAS RNA spanning nts 147–185 as the smallest region that still had affinity for IMP1 (S20 Fig). Based on these studies, we designed a 39-nt CD44 RNA spanning nts 2862–2900 and a 44-nt KRAS RNA spanning nts 142–185 for use in the FP assay. These RNAs were commercially synthesized and labeled with fluorescein at the 3’end. It was important to first establish the validity of our FP assay. Using gel shift assay, we previously showed that IMP1 variant KH1-2 with point mutation at the GXXG motif in KH1 and KH2 domains had completely lost the ability to bind to CD44 RNA while the KH3-4 variant had binding affinity comparable to that of the WT [23]. KH3 variant, a single point mutation at the GXXG motif, had significantly reduced affinity for CD44 RNA [23]. As shown in Fig 7A, the relative affinity of KH1-2, KH3-4 and KH3 variants for the 39-nt fluorescein-labeled CD44 RNA as compared to the WT IMP1 was remarkably similar to that of gel shift assay. The KH1-2 variant did not increase fluorescence anisotropy (FA) units, even at 1000 nM, while the KH3 variant showed significantly reduced FA units compared to the WT. In the FP assay, we found that the KH3-4 variant had slightly reduced affinity for CD44 RNA compared to the WT IMP1 (Fig 7A). The small deviation could be attributed to the fact that a larger 194-nt CD44 RNA was used in the gel shift assay [23]. To further validate our FP assay, we used the 44-nt KRAS RNA. In the gel shift assay, we found that all the KH variants, single or double point mutations, had significantly reduced or lost the ability to bind to KRAS RNA [24,25]. Fig 7B shows that KH1-2 variant had no affinity while KH3-4 and KH3 variants had significantly reduced ability to bind to the 44-nt KRAS RNA as determined by FP. Again, this is consistent with the results from the gel shift assay [24,25]. In summary, we have successfully established a reliable FP assay that recapitulates the gel shift assay and therefore can be conveniently used to study the IMP1-RNA interaction.

### Confluentin inhibits IMP1-KRAS RNA interaction

**Fig 8:**
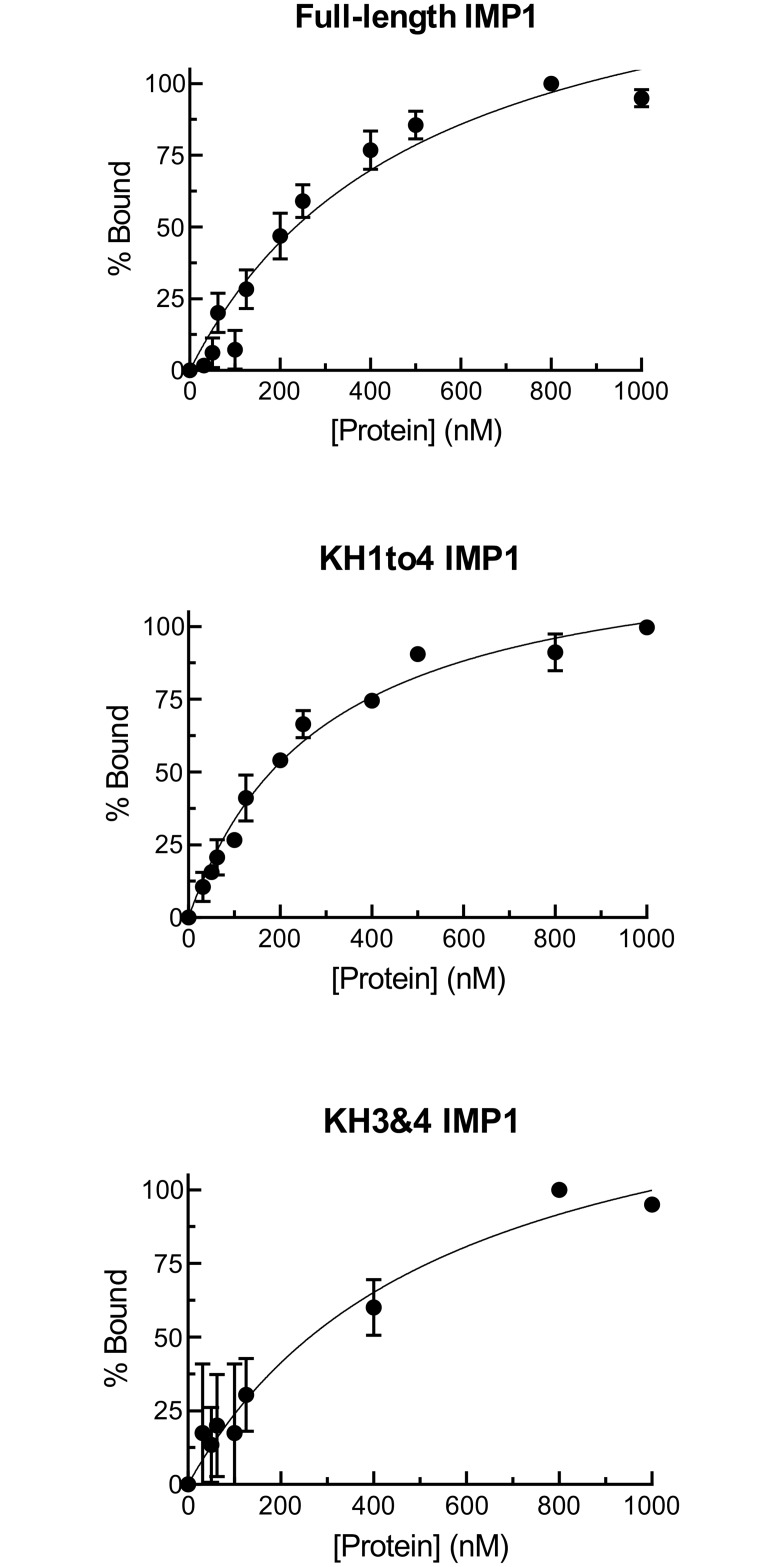
Binding curves of the full-length and truncated IMP1 to KRAS RNA as determined using fluorescence polarization. Increasing amounts of the full-length IMP1 or truncated IMP1 (KH1to4 and KH3&4) were incubated with 44-nt fluorescein-labeled KRAS RNA at 37°C for 30 min. Results shown represent three replicate experiments (n = 3).

**Fig 9:**
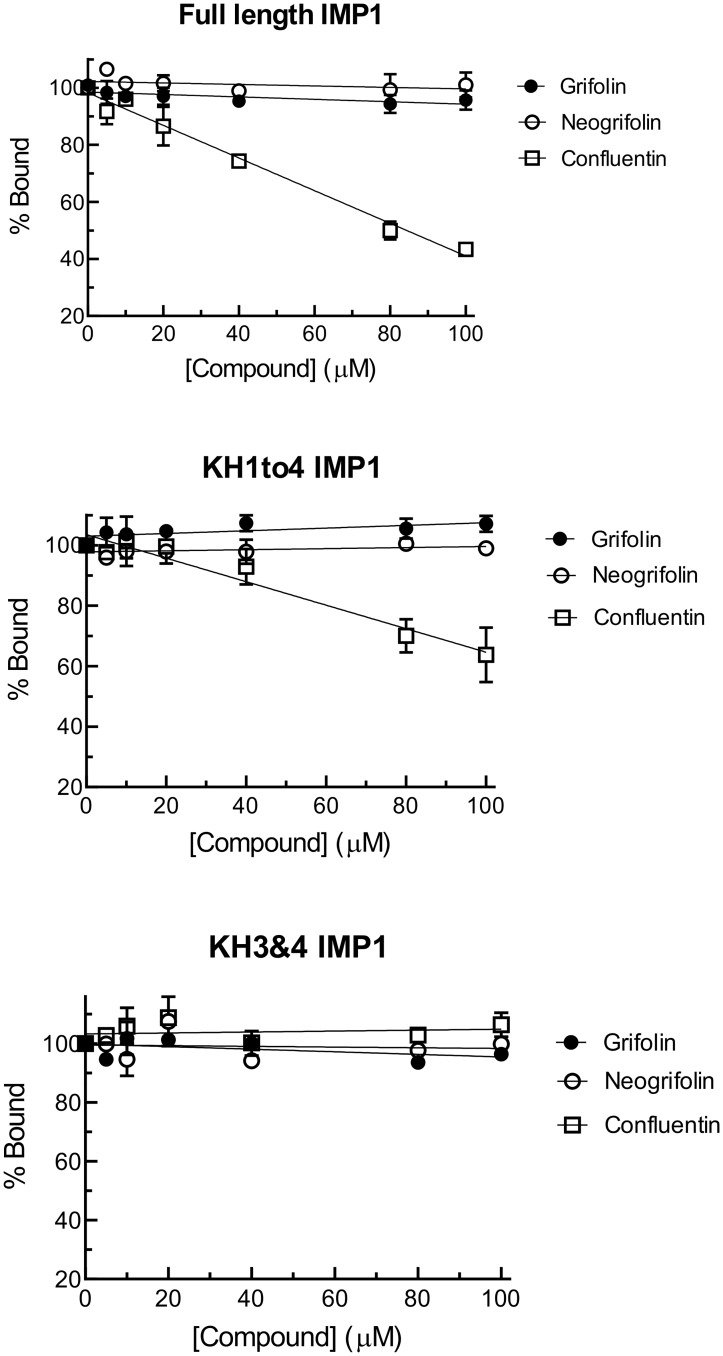
Effect of grifolin, neogrifolin and confluentin on the physical interaction between KRAS RNA and IMP1 protein. Recombinant IMP1 protein (300 nM) was incubated with fluorescently-labeled KRAS RNA in the absence or presence of various concentrations of grifolin, neogrifolin or confluentin. The concentrations of compounds used were: 100, 80, 40, 20, 10 and 5 μM. The three types of recombinant IMP1 proteins used were: full-length, KH1to4 and KH3&4. Data shown are mean ± S.D. pooled from three independent experiments (n = 3).

KRAS expression and cell survival in colon cancer cells can be regulated by binding IMP1 protein to KRAS mRNA [38]. Using the established FP assay, we showed that the full-length IMP1, truncated IMP1 containing only the KH1 to KH4 domains (KH1to4 IMP1), or only the KH3 and KH4 di-domain (KH3&4 IMP1), dose-dependently bind to 44-nt fluorescein-labeled KRAS RNA (Fig 8). We then assessed the dose-dependent effect of grifolin, neogrifolin and confluentin on fluorescein-labeled KRAS RNA bound to the three forms of recombinant IMP1. We determined that, for grifolin and neogrifolin, up to 100 μM had no effect on IMP1-KRAS RNA interaction (Fig 9). In contrast, confluentin clearly inhibited the interaction between KRAS RNA and the full-length IMP1, starting at 40 μM. It also significantly inhibited the interaction between KRAS RNA and KH1to4 IMP1 at concentrations above 40 μM (Fig 9). Surprisingly, confluentin had no effect on the KRAS RNA-IMP1 KH3&4 interaction (Fig 9), suggesting that confluentin binds to site(s) on IMP1 that are outside of the KH3&4 di-domain. Given that the only difference between KH1to4 IMP1 and KH3&4 IMP1 is the presence of KH1 and KH2 domains in the former, it is highly likely that confluentin binds to the KH1&2 di-domain to exert its inhibitory effect on IMP1-KRAS RNA interaction. The KH1 and KH2 di-domain has been shown to be critical for IMP1 to bind RNA substrates [23,25,41,42], including the KRAS RNA [24,25]. In addition, a recent study suggested that the KH1&KH2 di-domain of IMP1 has high RNA-binding affinity and binds RNA rapidly [43]. Further study is clearly required to test the hypothesis that confluentin binds to KH1 and KH2 di-domain to inhibit the function of IMP1.

**Fig 10:**
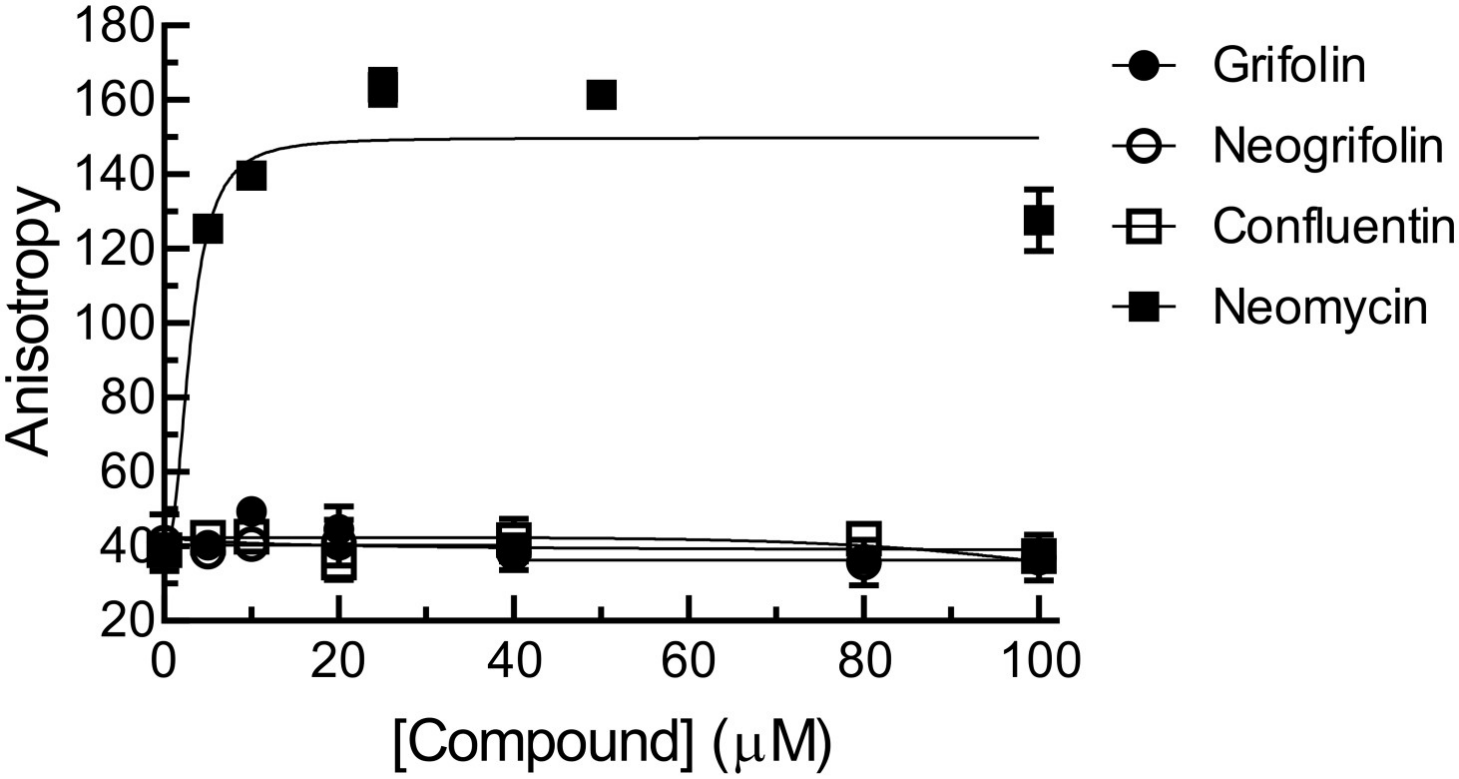
Grifolin (1), neogrifolin (2) and confluentin (3) do not bind to KRAS RNA. Increasing concentrations of grifolin, neogrifolin and confluentin were incubated with the 44-nt fluorescein-labeled KRAS RNA at 37°C for 30 min. Fluorescence anisotropy units were then measured. Neomycin was used as the positive control. The results shown represent three replicate experiments (n = 3).

When incubated with the 44-nt fluorescein-labeled KRAS RNA alone, grifolin, neogrifolin and confluentin had no effect on fluorescence anisotropy units (Fig 10), indicating that these compounds have no affinity for KRAS RNA. However, the antibiotic neomycin, known to bind RNA [44,45], significantly enhanced fluorescence anisotropy units when incubated with the fluorescently-labeled KRAS RNA (Fig 10). This result supports the theory that confluentin inhibits IMP1-KRAS RNA interaction by targeting IMP1 protein. To our knowledge, this is the first description of a small molecule inhibiting IMP1-KRAS RNA interaction. A small molecule called BTYNB has been reported to inhibit IMP1-c-*myc* RNA interaction [46]. The present study raises the possibility that confluentin suppresses KRAS expression by disrupting IMP1-KRAS mRNA in colon cancer cells. As such, it would be important to determine whether confluentin can inhibit other IMP1-RNA interactions, leading to the potential functional inhibition of IMP1 in cell lines and in animal models.

It is important to point out the limitation of the fluorescence polarization method used here: only a small fragment of KRAS mRNA (44-nt) that directly interacted with IMP1 was studied. In a two-dimensional cell culture and three-dimensional animal model, such interaction involving the entire KRAS mRNA (about 5,400-nt) is more complex and most likely involves many other ribonucleoproteins. Nevertheless, our findings that confluentin specifically inhibits KRAS RNA-IMP1 interaction and KRAS expression in human colon cancer cells are worthy of further investigations. For instance, it would be of interest to determine whether confluentin is able to inhibit IMP1 granule formation in zebrafish embryos [23], an early event in RNA-IMP1 interaction *in vivo*. It is also important to determine the mechanism whereby the three compounds inhibit KRAS expression in human colon cancer cells, and whether they have an effect on KRAS expression in pancreatic and lung cancer cells as well as cholangiocarcinoma cells where KRAS is highly implicated.

## Conclusions

The results from this study show that the three major anti-cell viability compounds from the ethanol extract of the mushroom *A*. *flettii*, collected in north-central BC, are grifolin, neogrifolin and confluentin. We also demonstrate for the first time that confluentin induces apoptosis and arrests the cell cycle at the G2/M phase in colon cancer cells. Grifolin, neogrifolin and confluentin suppress KRAS expression in colon cancer cells, a novel bioactivity for these compounds. In this study, we described an *in vitro* fluorescence polarization method to conveniently study KRAS RNA-IMP1 interaction. Using this method, we found that confluentin specifically inhibit the physical interaction between IMP1 and KRAS RNA. As such, it remains important to consider further the exploration of confluentin as a potential inhibitor of the oncogenic function of IMP1 in animal cells.

## Supporting information

S1 Fig HPLC UV spectrum showing the relative abundance and retention times of the three major peaks at λ_max_ 279 nm. An analytical column Agilent Zorbax Eclipse XBD-C18 (4.6 mm x 150 mm) was used.
(TIF)

S2 Fig HPLC-chromatogram (monitored at 279 nm), and MS-spectra of grifolin. The ESIMS spectrum exhibited a [M + H]^+^ peak at *m/z* = 329.3.
(TIF)

S3 Fig ^1^H-NMR spectrum of grifolin (CDCl_3_ with 0.3% TMS, 300 MHz). (TIF)

S4 Fig ^13^C-NMR spectrum of grifolin (1) (CDCl_3_ with 0.3% TMS, 300 MHz). (TIF)

S5 Fig COSY spectrum of grifolin (1) (CDCl_3_ with 0.3% TMS, 300 MHz). (TIF)

S6 Fig HSQC spectrum of grifolin (1) (CDCl_3_ with 0.3% TMS, 300 MHz). (TIF)

S7 Fig HPLC-chromatogram (monitored at 279 nm), and MS-spectra of neogrifolin. The ESIMS spectrum exhibited a [M + H]^+^ peak at *m/z* = 329.3.
(TIF)

S8 Fig ^1^H-NMR spectrum of neogrifolin (2) (CDCl_3_ with 0.3% TMS, 300 MHz). (TIF)

S9 Fig ^13^C-NMR spectrum of neogrifolin (2) (CDCl_3_ with 0.3% TMS, 300 MHz). (TIF)

S10 Fig COSY spectrum of neogrifolin (CDCl_3_ with 0.3% TMS, 300 MHz). (TIF)

S11 Fig HSQC spectrum of neogrifolin (CDCl3 with 0.3% TMS, 300 MHz). (TIF)

S12 Fig HPLC-chromatogram (monitored at 279 nm), and MS-spectra of confluentin. The ESIMS spectrum exhibited a [M + H]^+^ peak at *m/z* = 327.2.
(TIF)

S13 Fig ^1^H-NMR spectrum of confluentin (CDCl3 with 0.3% TMS, 300 MHz). (TIF)

S14 Fig ^13^C-NMR spectrum of confluentin (CDCl3 with 0.3% TMS, 300 MHz). (TIF)

S15 Fig COSY spectrum of confluentin (CDCl3 with 0.3% TMS, 300 MHz). (TIF)

S16 Fig HSQC spectrum of confluentin (CDCl3 with 0.3% TMS, 300 MHz). (TIF)

S17 Fig HSQC spectrum of confluentin (expanded: 4.8–7.0 ppm) (CDCl3 with 0.3% TMS, 300 MHz). (TIF)

S18 Fig HSQC spectrum of confluentin (expanded: 0–3.0 ppm) (CDCl3 with 0.3% TMS, 300 MHz). (TIF)

S19 Fig Effect of grifolin, neogrifolin, and confluentin on KRAS expression in HT29 human colon cancer cells. HT29 cells were treated with 20 μM (A) or 50 μM (A and B) of grifolin, neogrifolin and confluentin for 48 hours. Isolated cell lysates were then subjected to Western blot analysis.
(TIF)

S20 Fig Mapping the IMP1 binding site on KRAS RNA using electrophoretic mobility shift assay. **(A)** A summary of 3’ and 5’ end truncated KRAS RNA fragments corresponding to nts 1–185, and their relative binding affinity for IMP1. **(B)** Electrophoretic mobility shift assay of 3’ and 5’ end truncated KRAS RNA fragments corresponding to nts 1–185. **(C)** A summary of 3’ and 5’ end truncated KRAS RNA fragments corresponding to nts 93–185, and their relative binding affinity for IMP1. **(D)** Electrophoretic mobility shift assay of 3’ and 5’ end truncated KRAS RNA fragments corresponding to nts 93–185.
(TIF)

S1 Table ^13^C-NMR spectral data of purified grifolin, neogrifolin and confluentin compared to published data. (DOCX)

S2 Table ^1^H-NMR spectral data of purified grifolin, neogrifolin and confluentin compared to published data. (DOCX)

S1 Raw Images (PDF)
