# Supplementary figures and images for: Grifolin, neogrifolin and confluentin from the terricolous polypore Albatrellus flettii suppress KRAS expression in human colon cancer cells
Source: PLoS One. 2020 May 5;15(5):e0231948. doi: 10.1371/journal.pone.0231948 (PMC7199964; doi:10.1371/journal.pone.0231948)

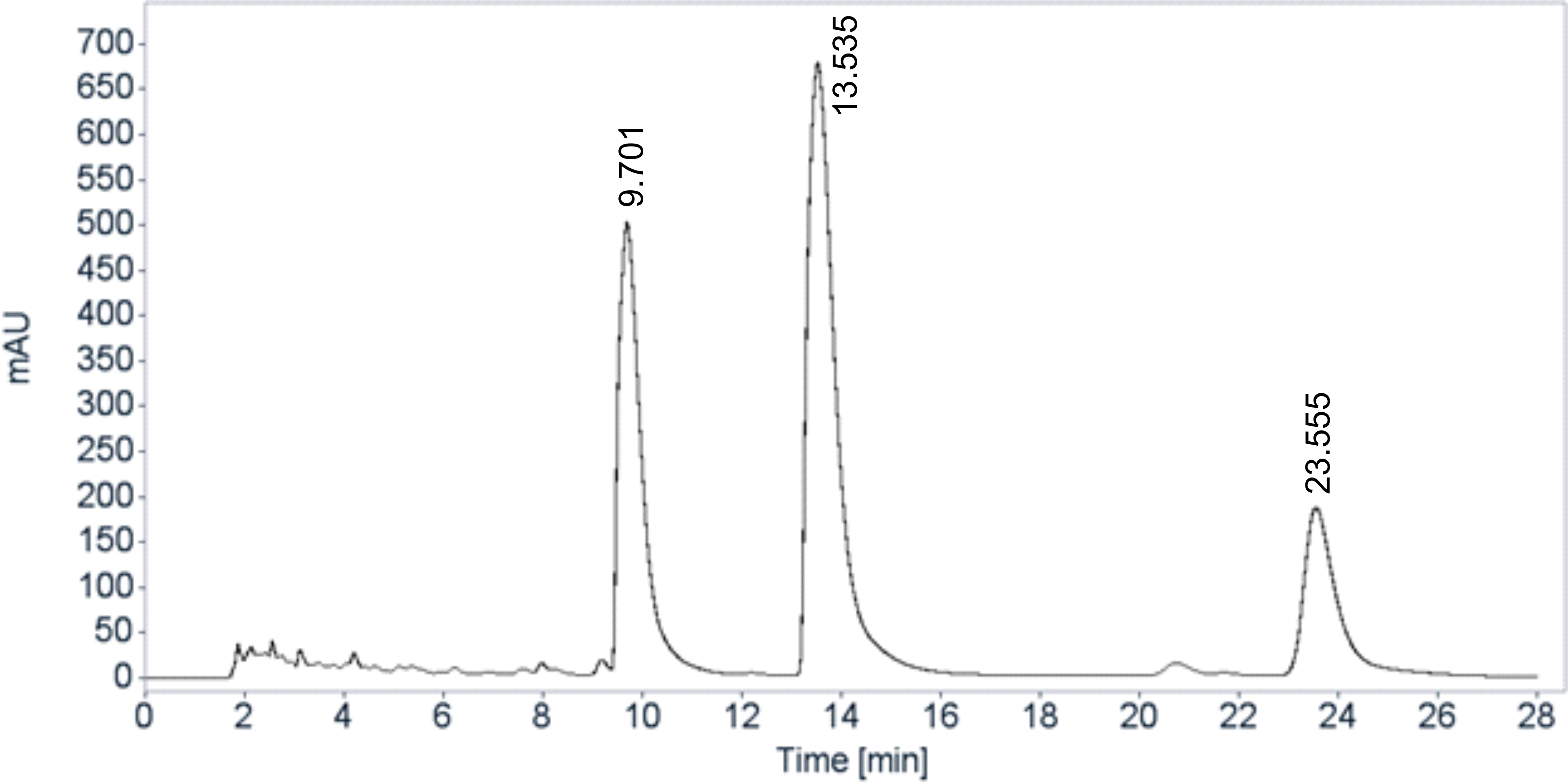

Supplement: S1 Fig — An analytical column Agilent Zorbax Eclipse XBD-C18 (4.6 mm x 150 mm) was used. (TIF) [file pone.0231948.s001.tif]

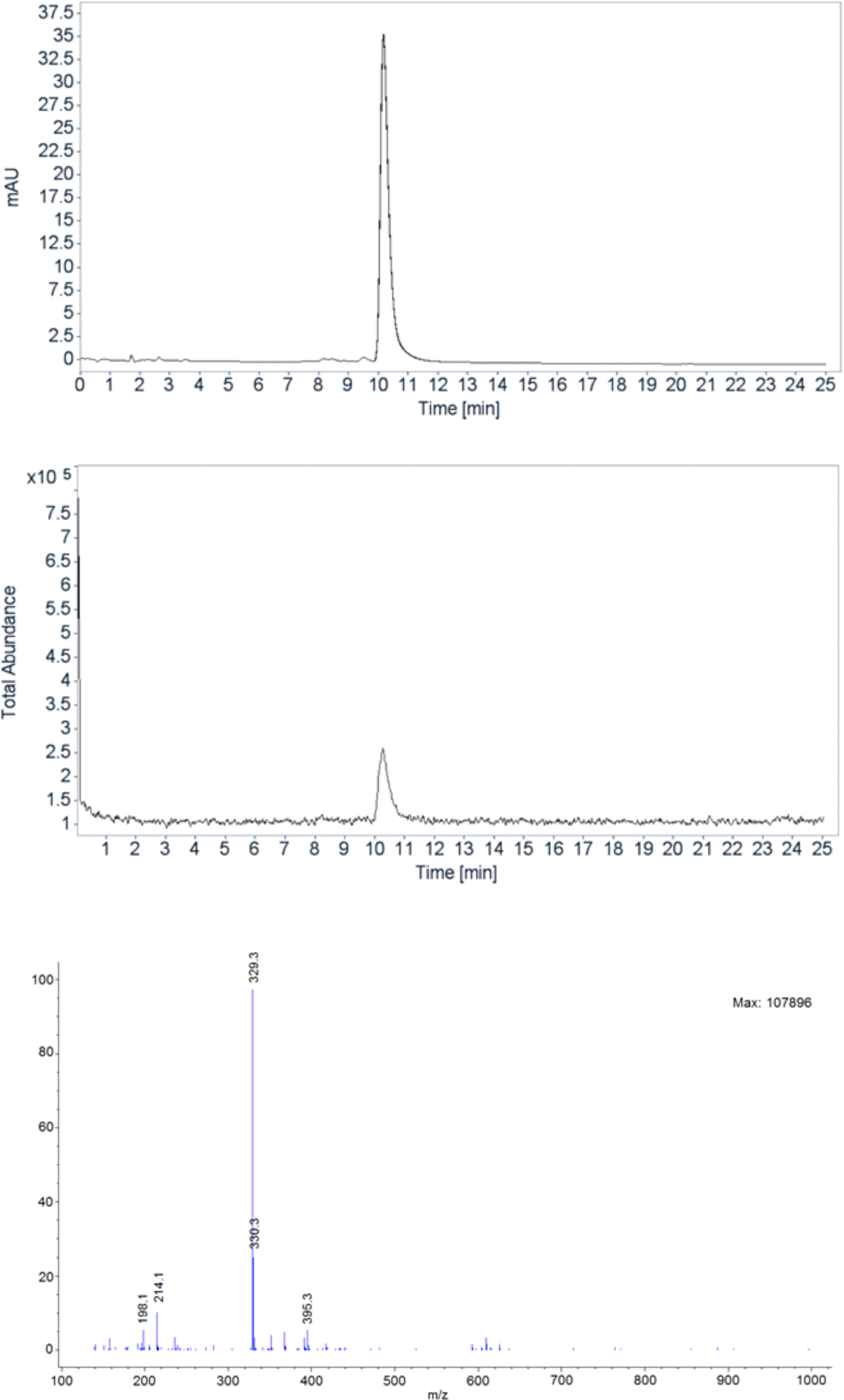

Supplement: S2 Fig — The ESIMS spectrum exhibited a [M + H]+ peak at m/z = 329.3. (TIF) [file pone.0231948.s002.tif]

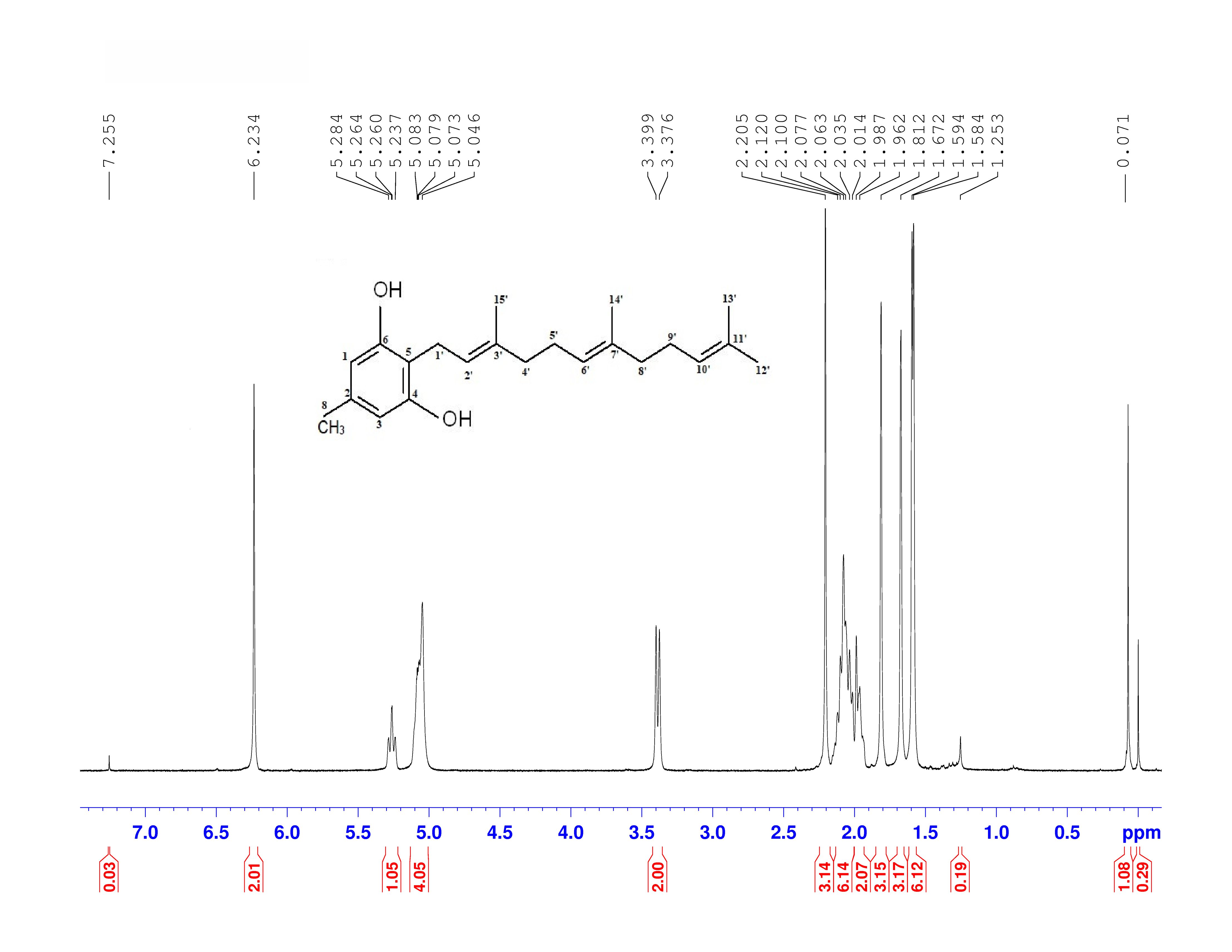

Supplement: S3 Fig — (TIF) [file pone.0231948.s003.tif]

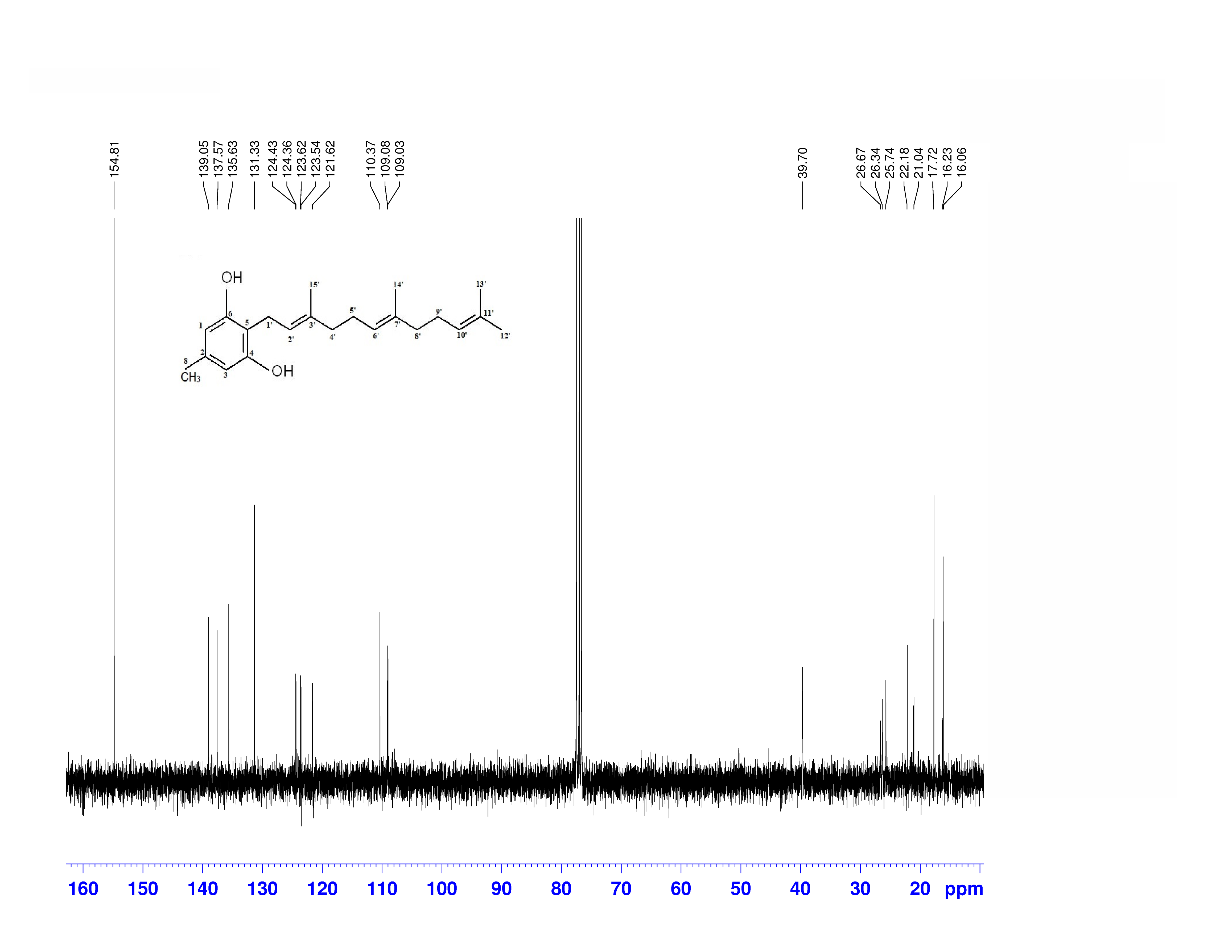

Supplement: S4 Fig — (TIF) [file pone.0231948.s004.tif]

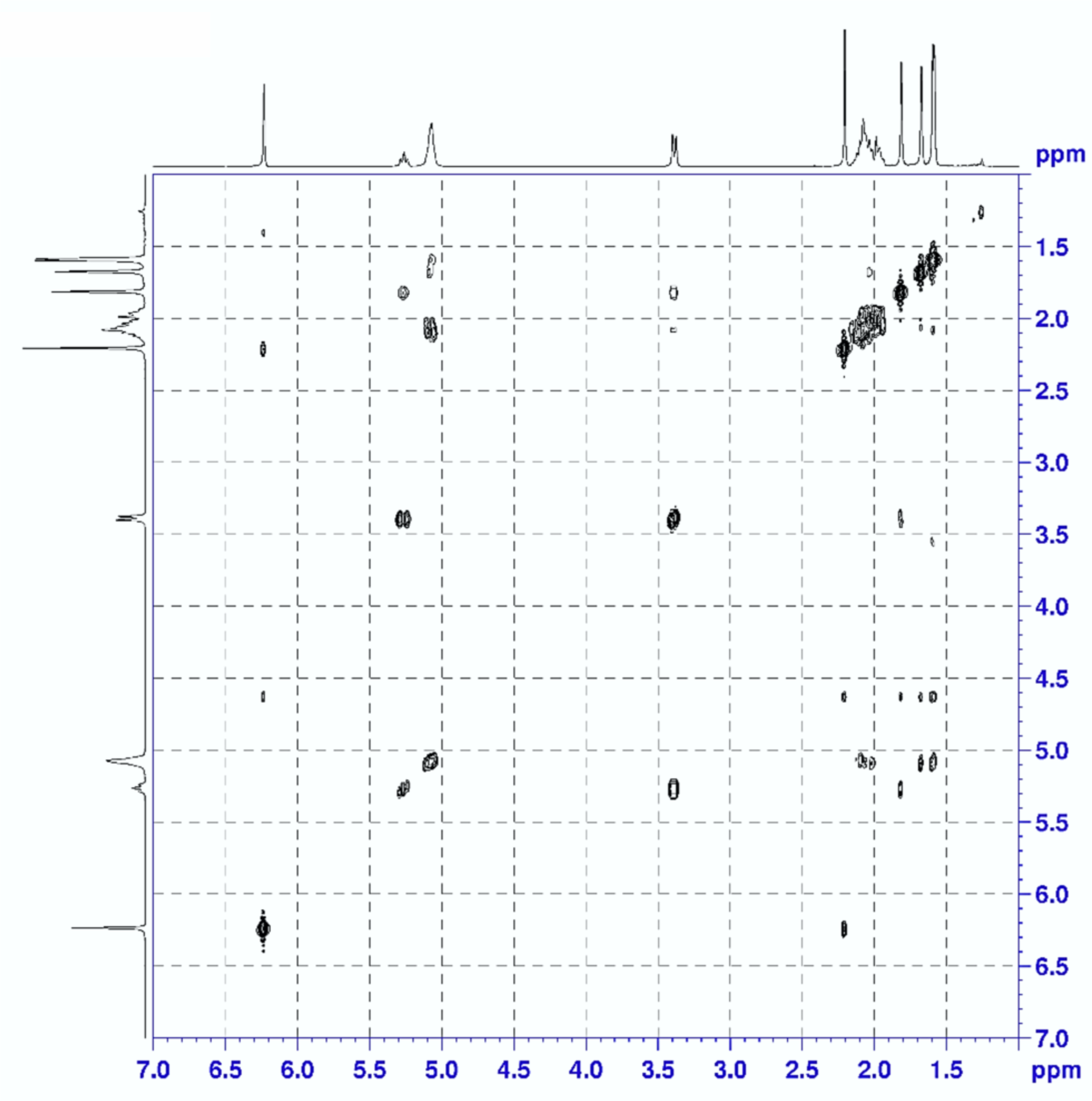

Supplement: S5 Fig — (TIF) [file pone.0231948.s005.tif]

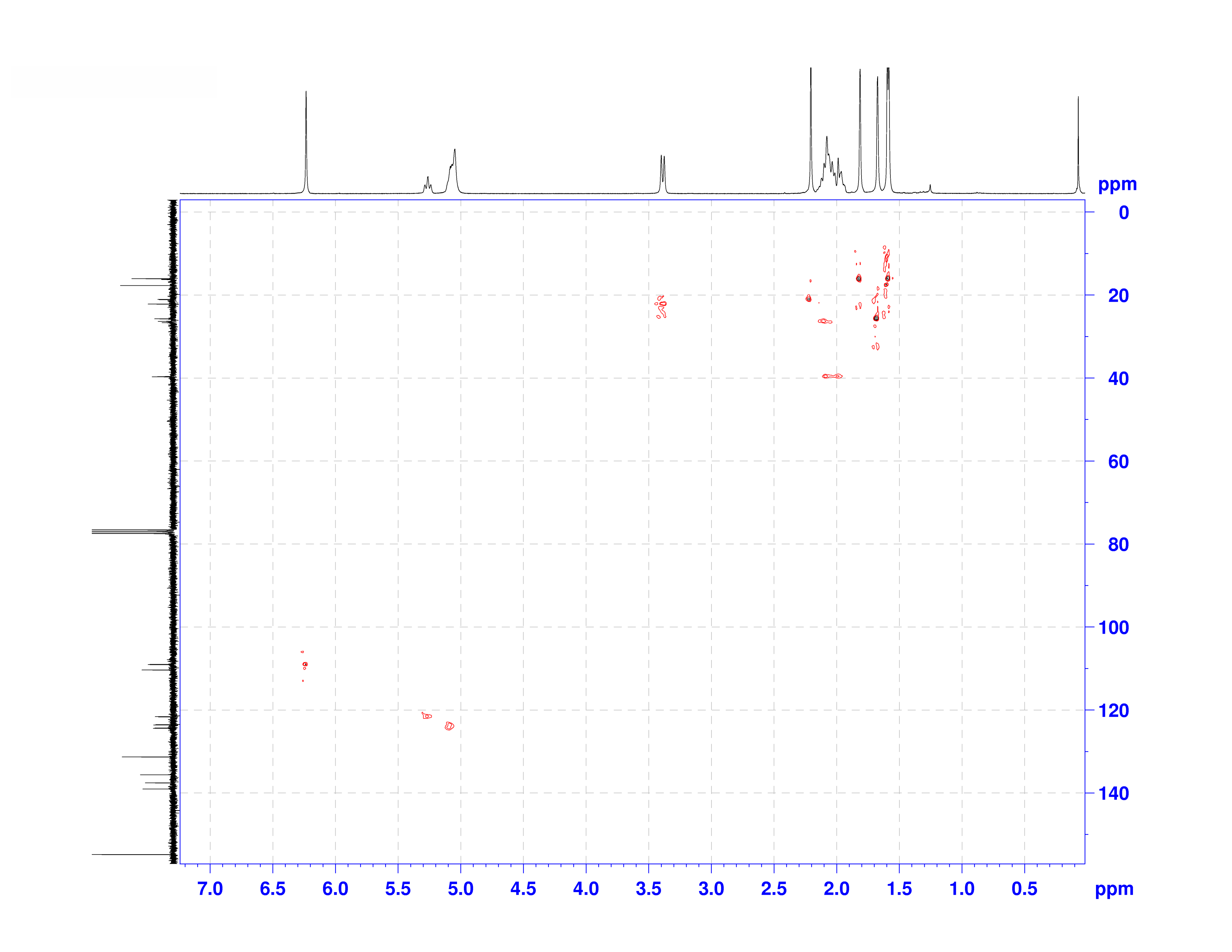

Supplement: S6 Fig — (TIF) [file pone.0231948.s006.tif]

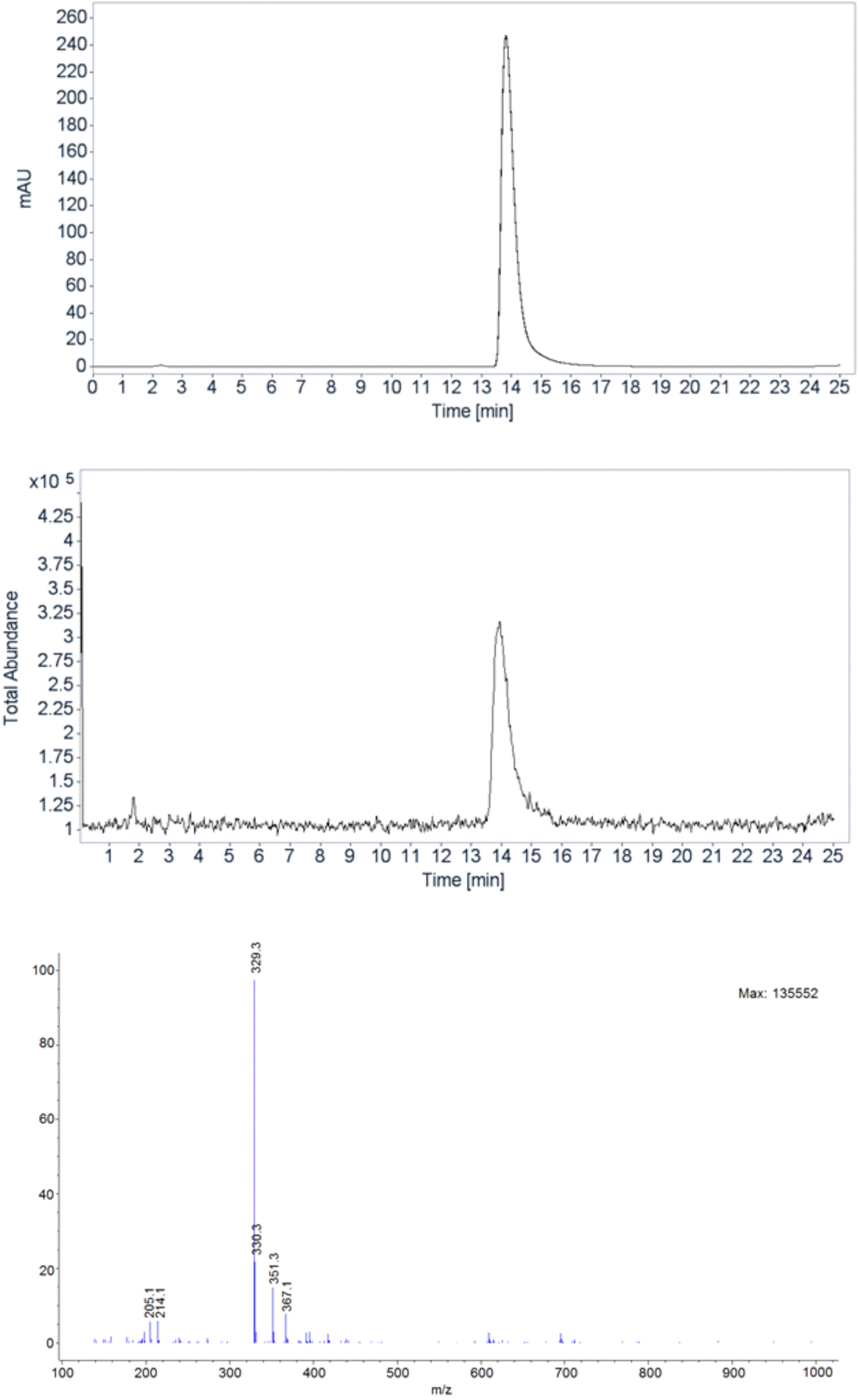

Supplement: S7 Fig — The ESIMS spectrum exhibited a [M + H]+ peak at m/z = 329.3. (TIF) [file pone.0231948.s007.tif]

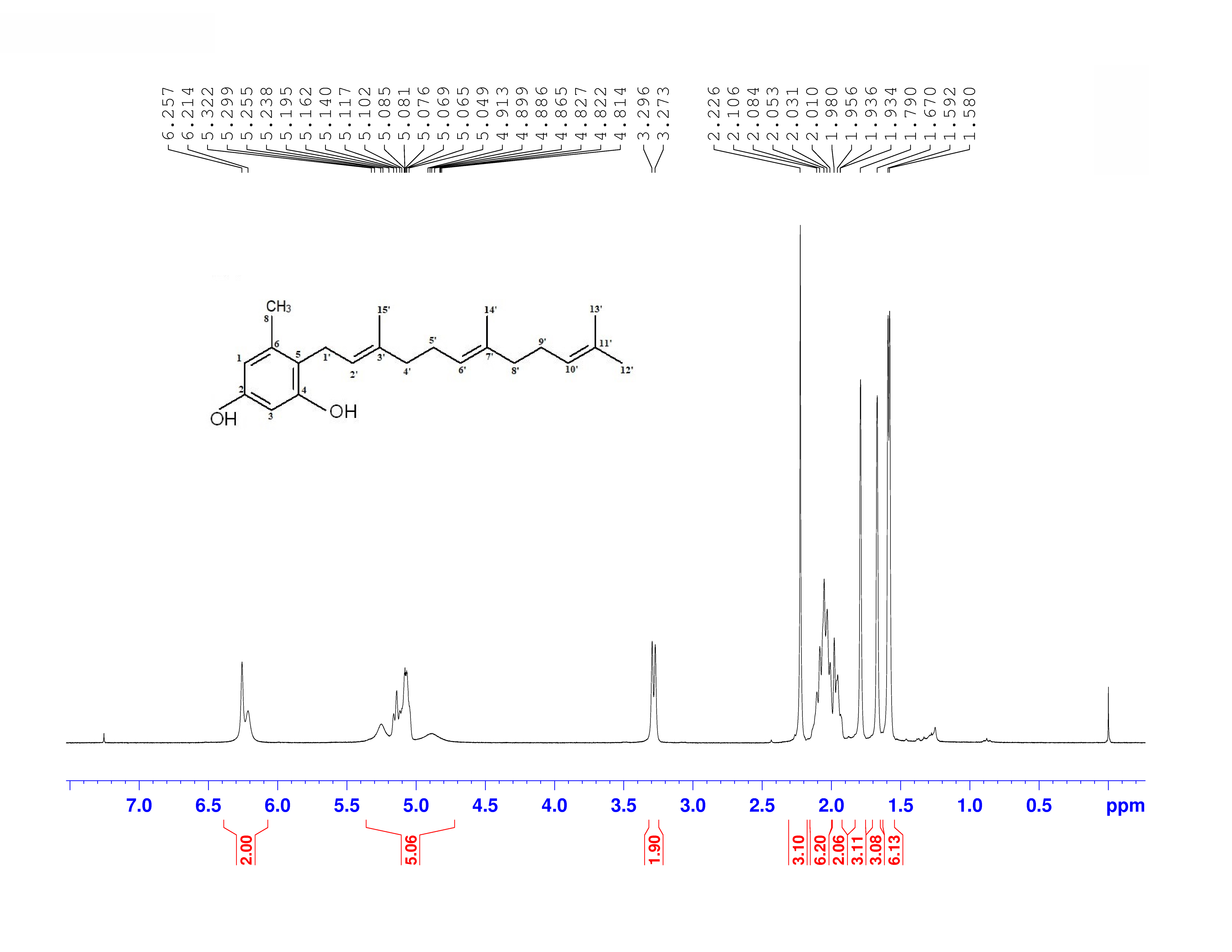

Supplement: S8 Fig — (TIF) [file pone.0231948.s008.tif]

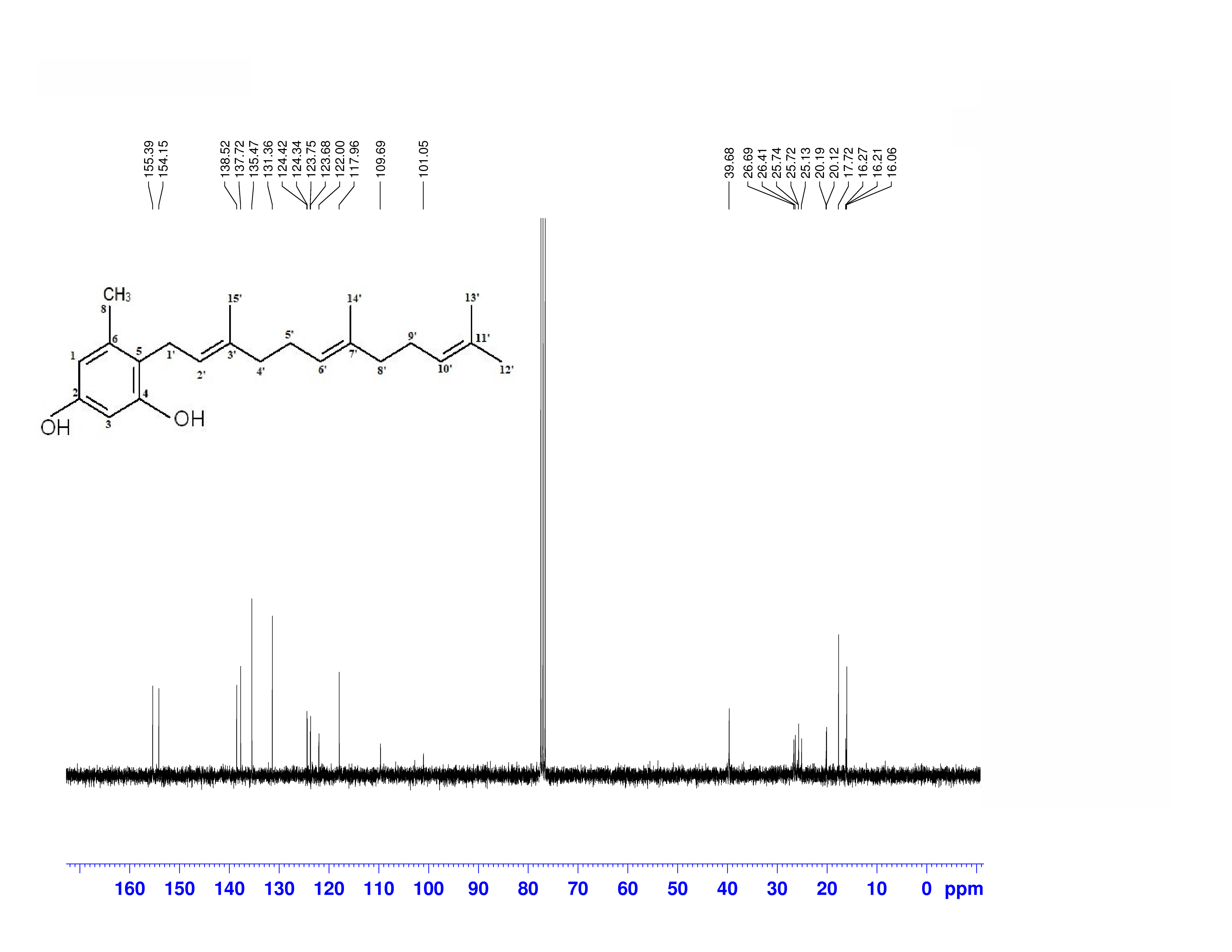

Supplement: S9 Fig — (TIF) [file pone.0231948.s009.tif]

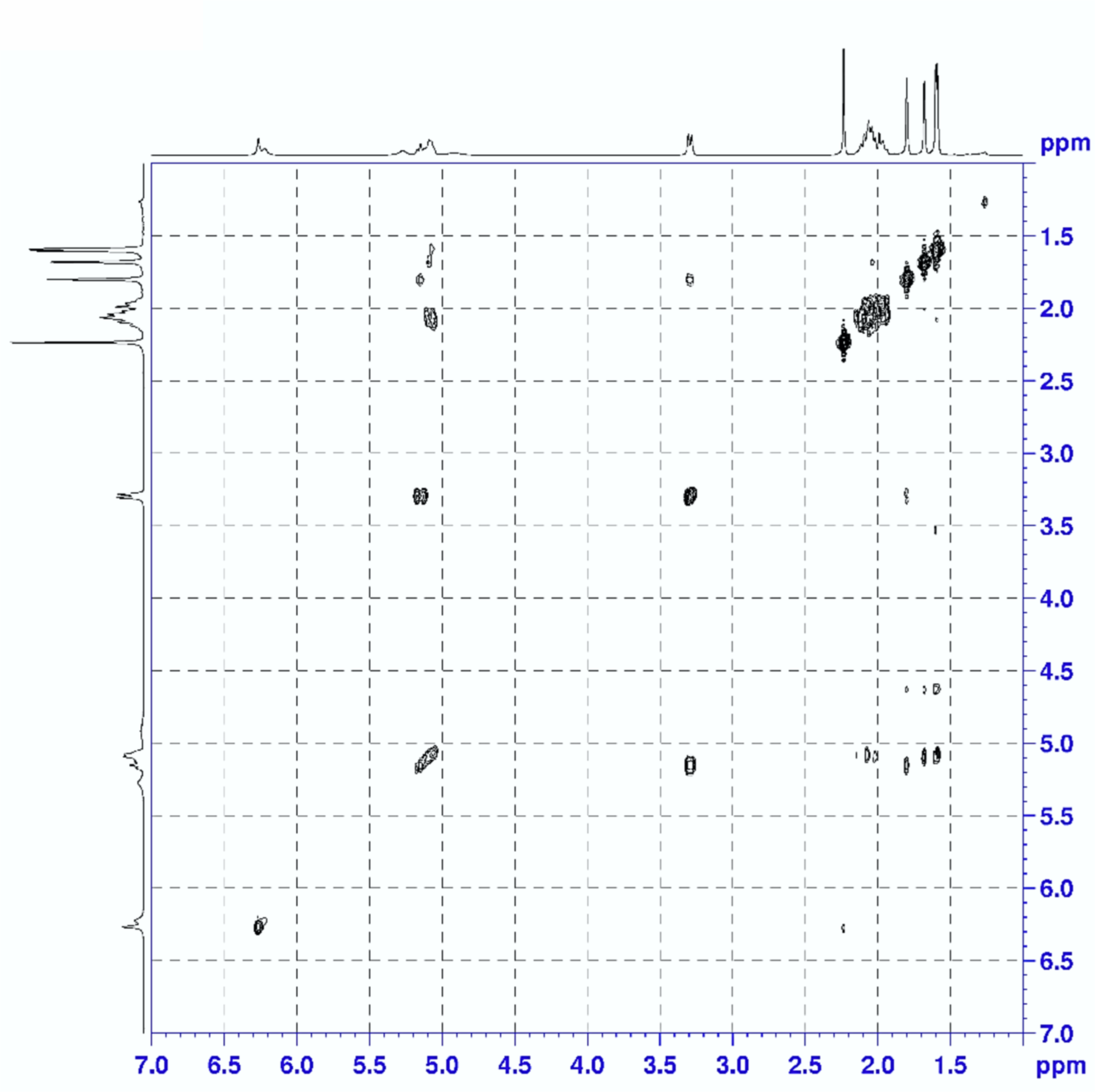

Supplement: S10 Fig — (TIF) [file pone.0231948.s010.tif]

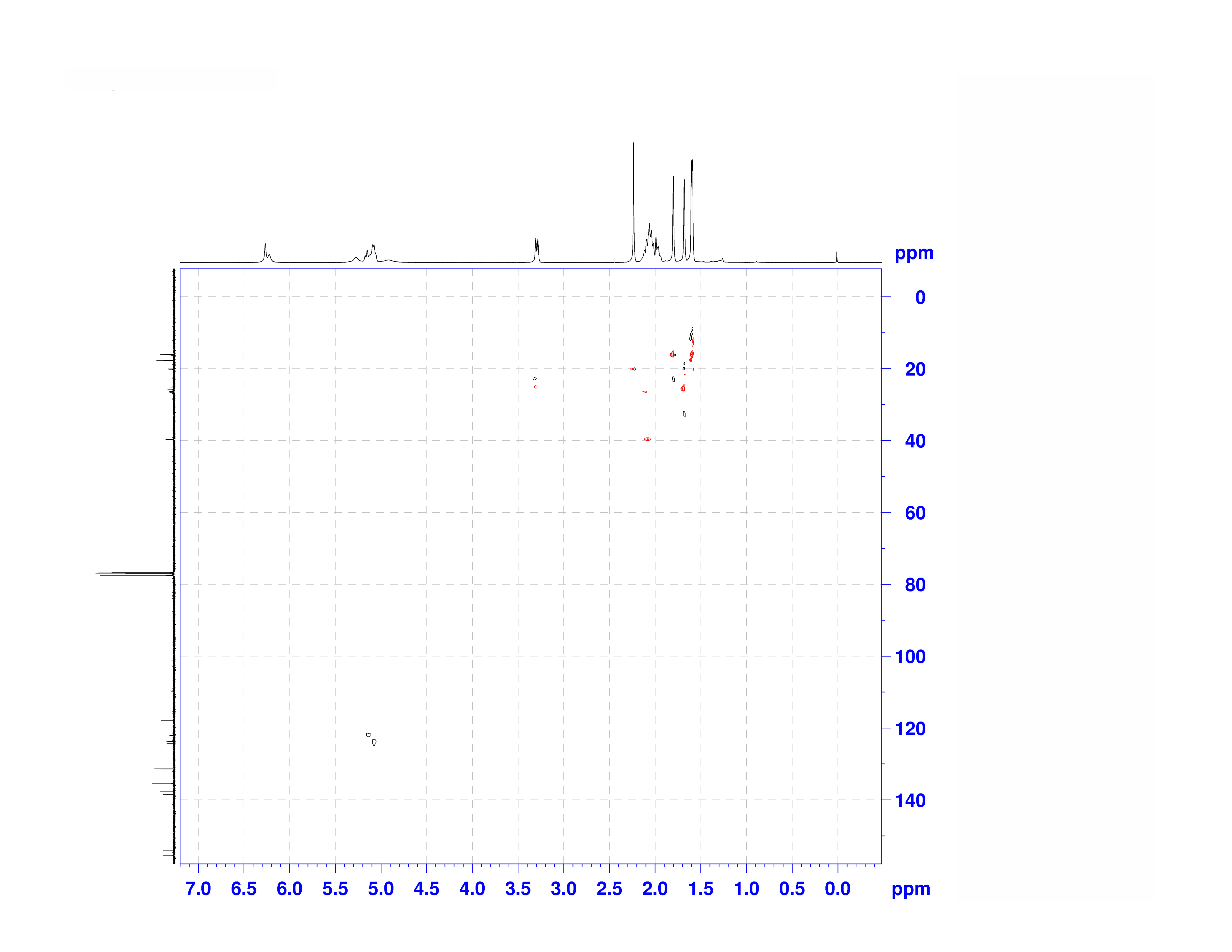

Supplement: S11 Fig — (TIF) [file pone.0231948.s011.tif]

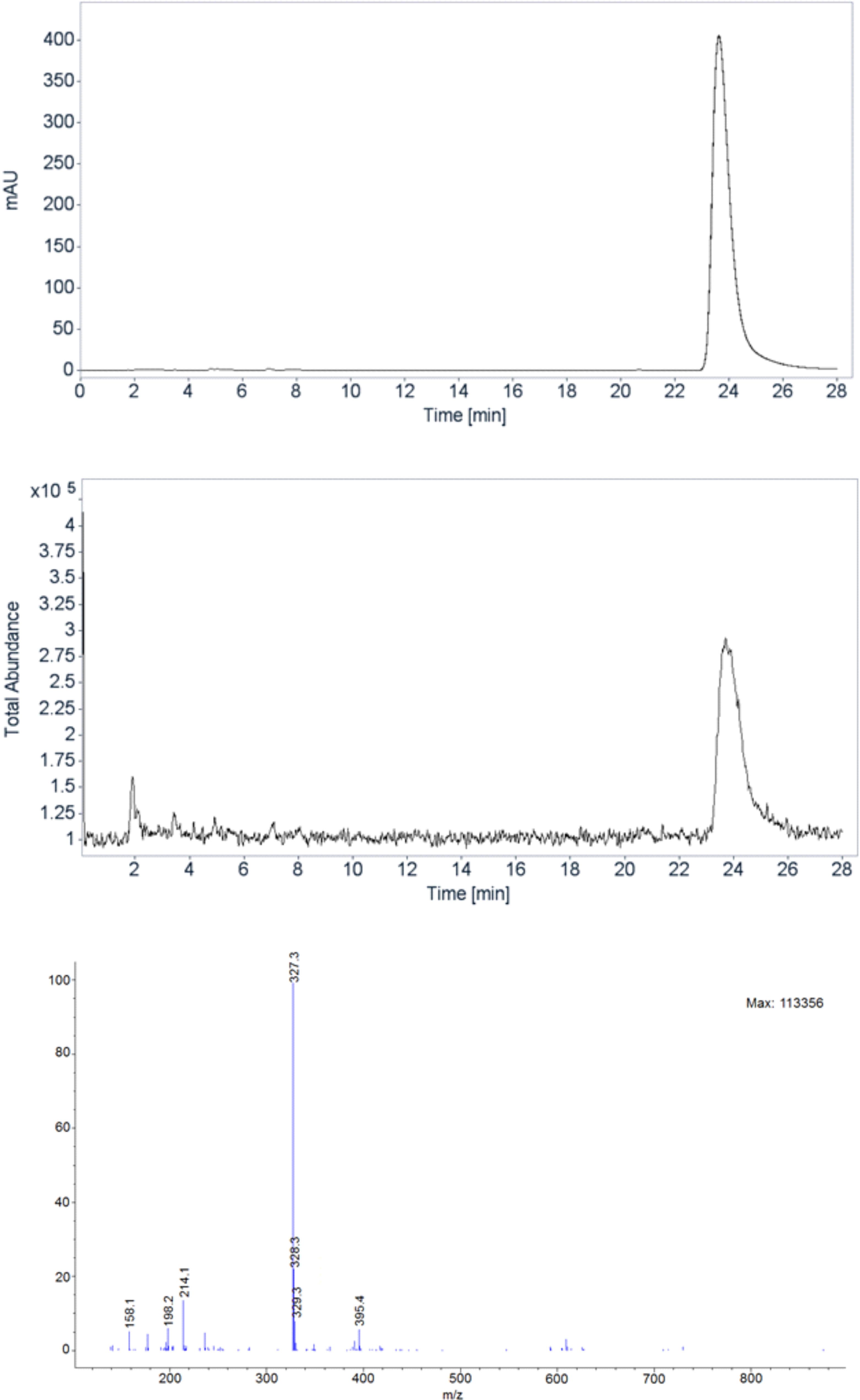

Supplement: S12 Fig — The ESIMS spectrum exhibited a [M + H]+ peak at m/z = 327.2. (TIF) [file pone.0231948.s012.tif]

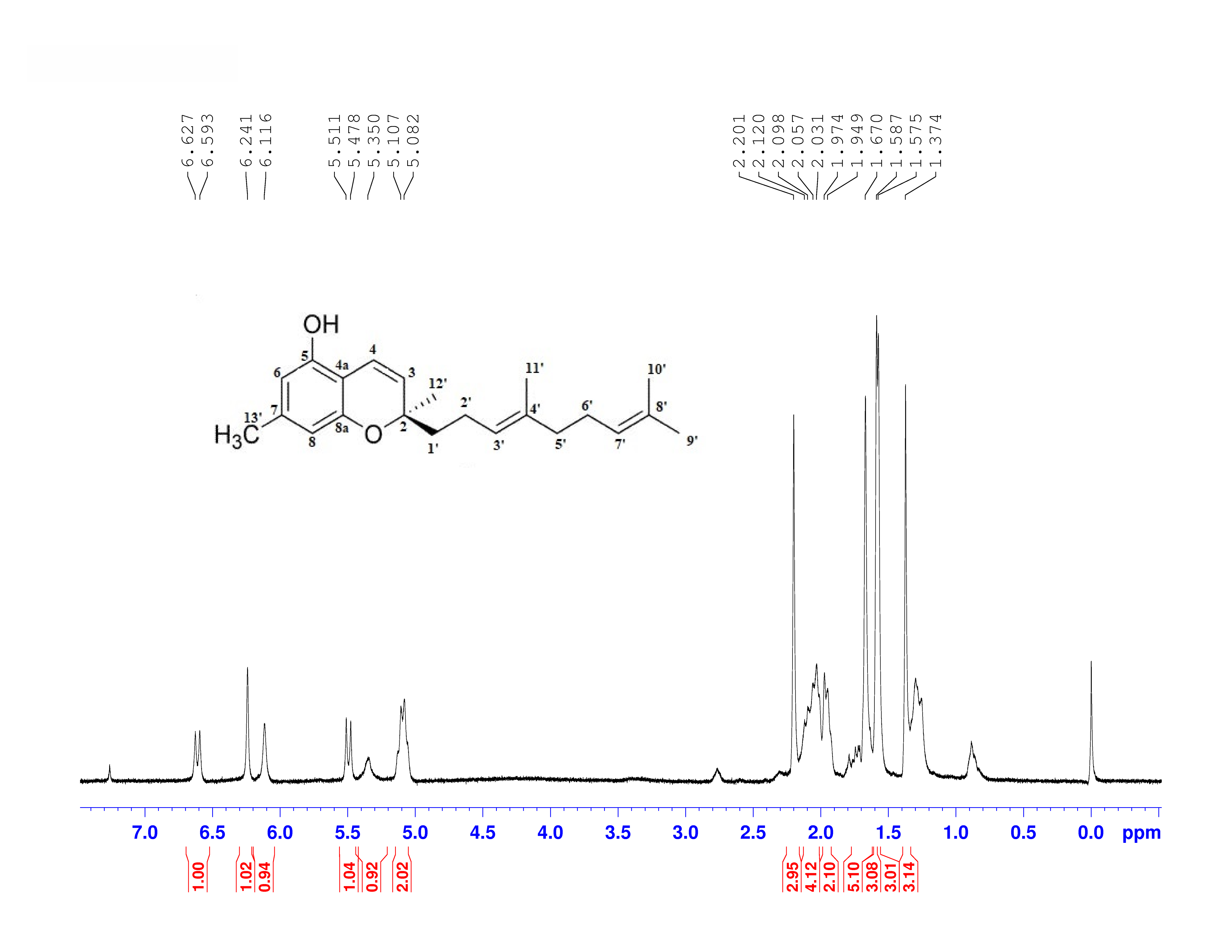

Supplement: S13 Fig — (TIF) [file pone.0231948.s013.tif]

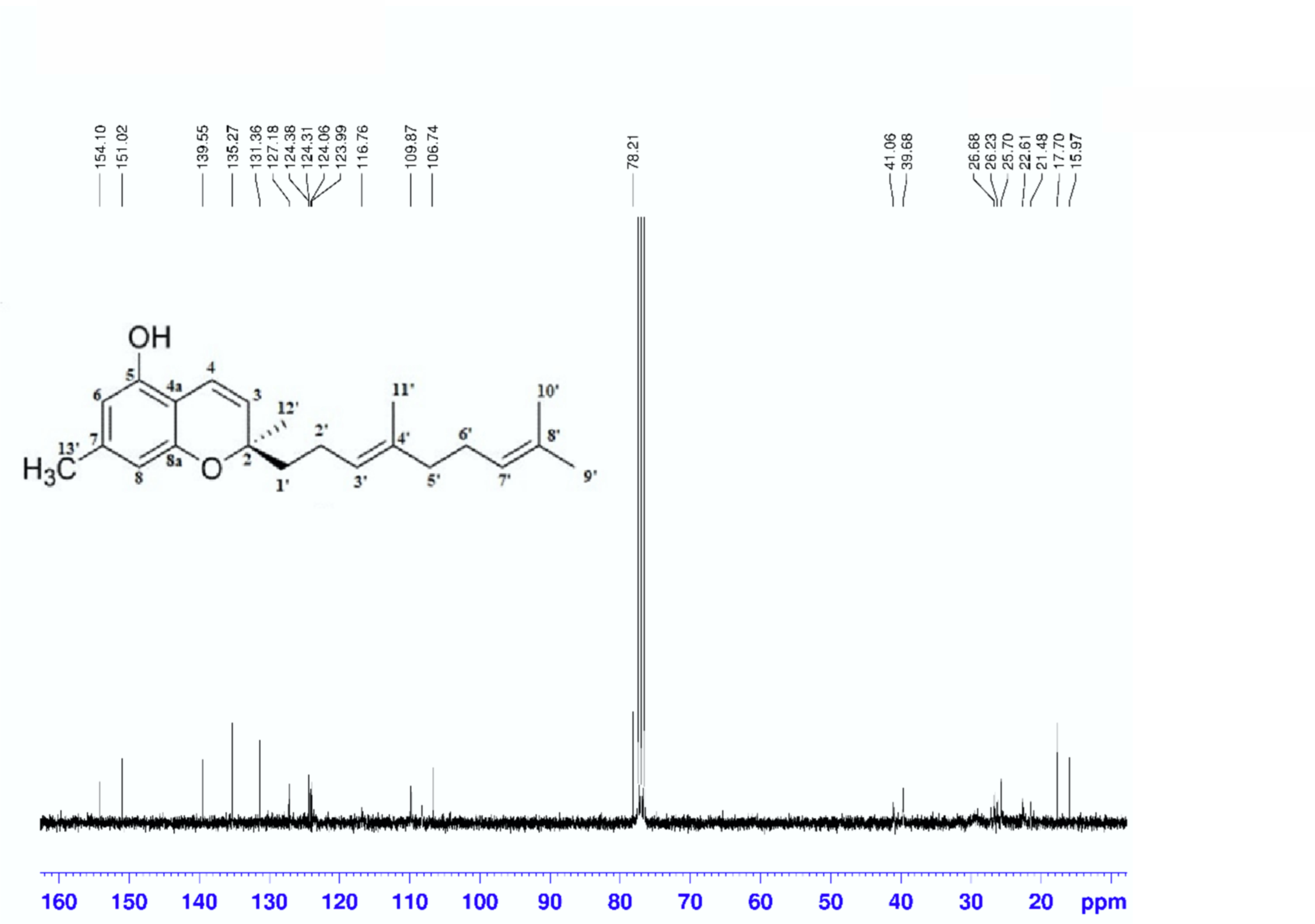

Supplement: S14 Fig — (TIF) [file pone.0231948.s014.tif]

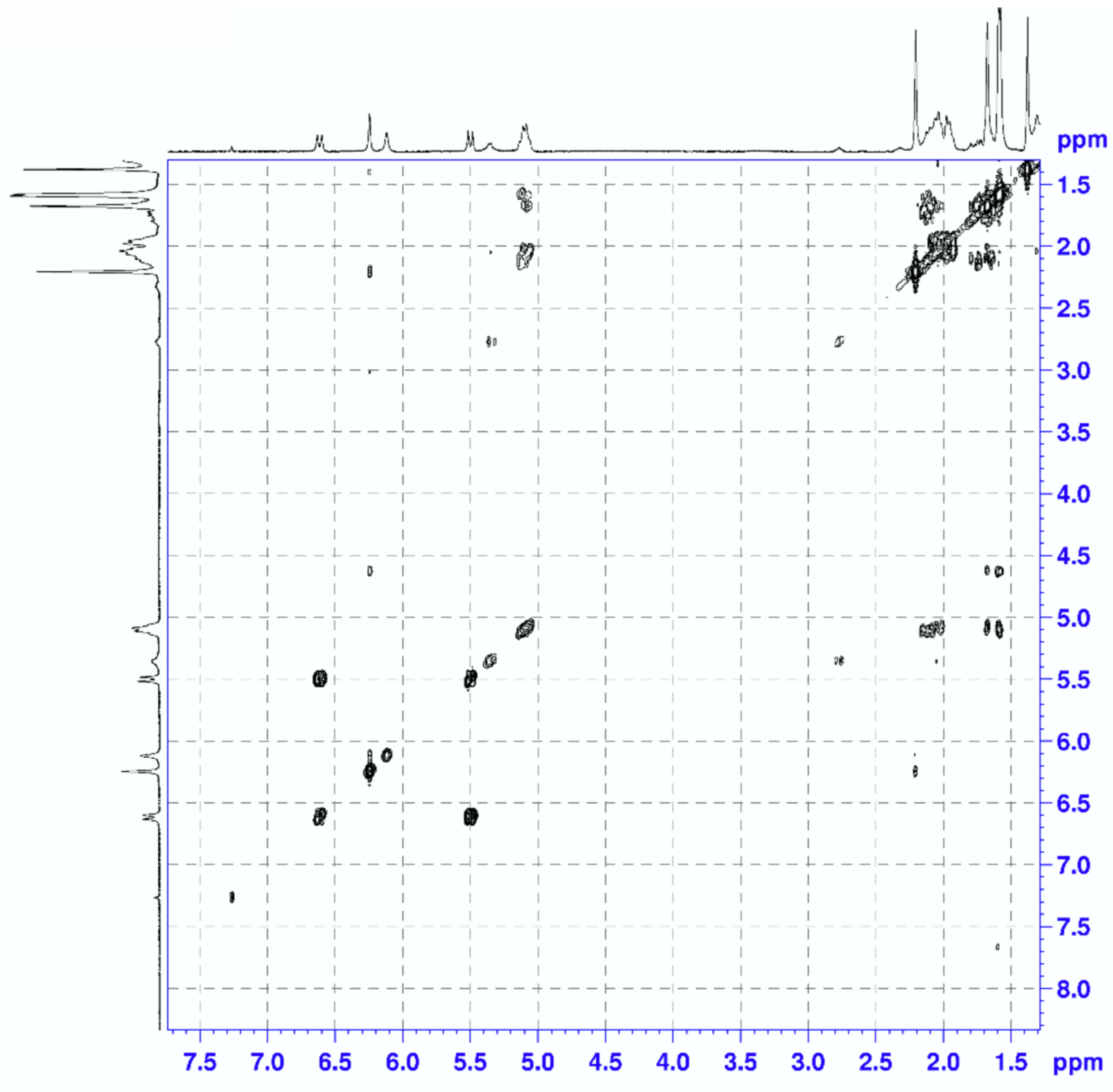

Supplement: S15 Fig — (TIF) [file pone.0231948.s015.tif]

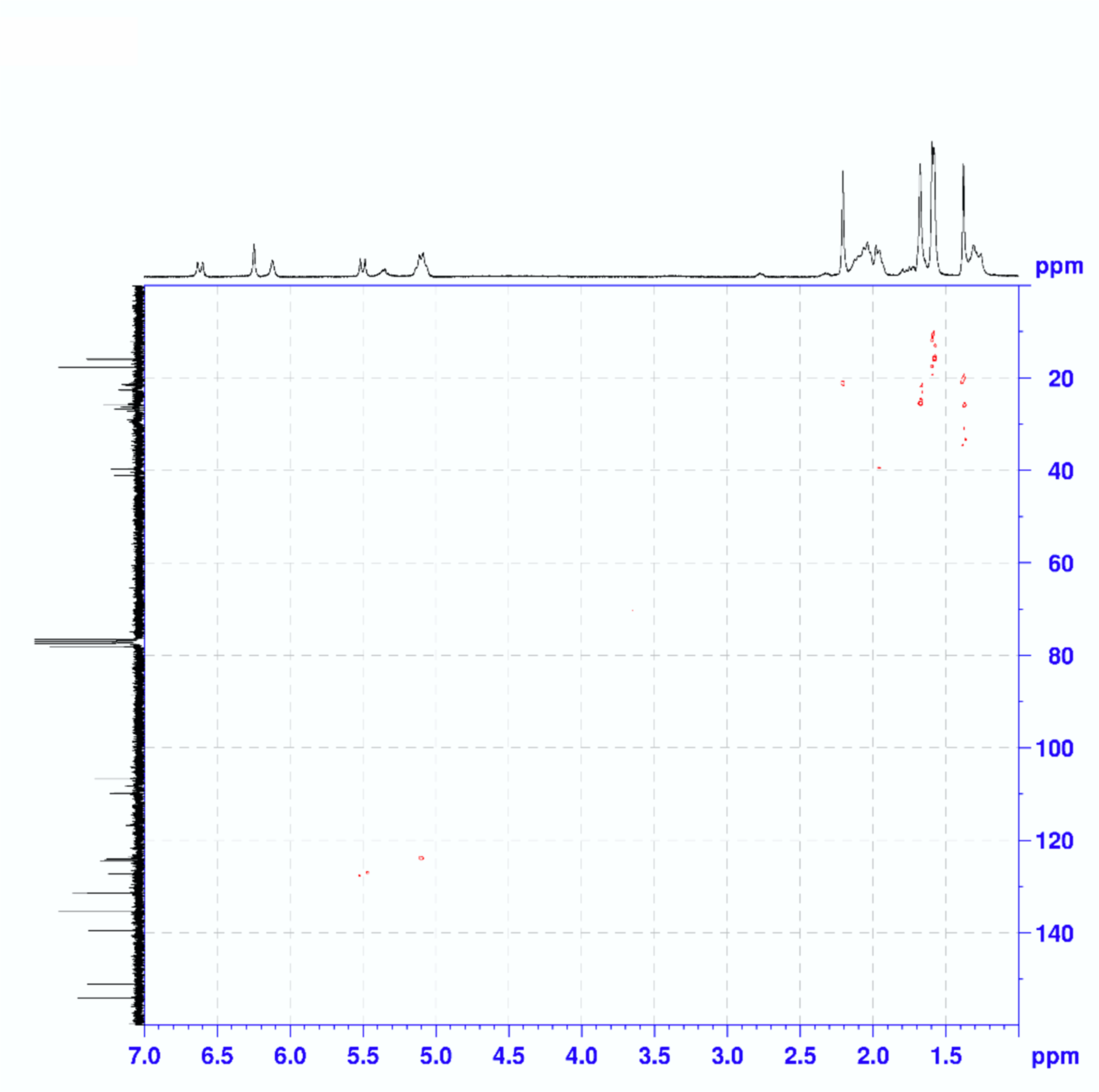

Supplement: S16 Fig — (TIF) [file pone.0231948.s016.tif]

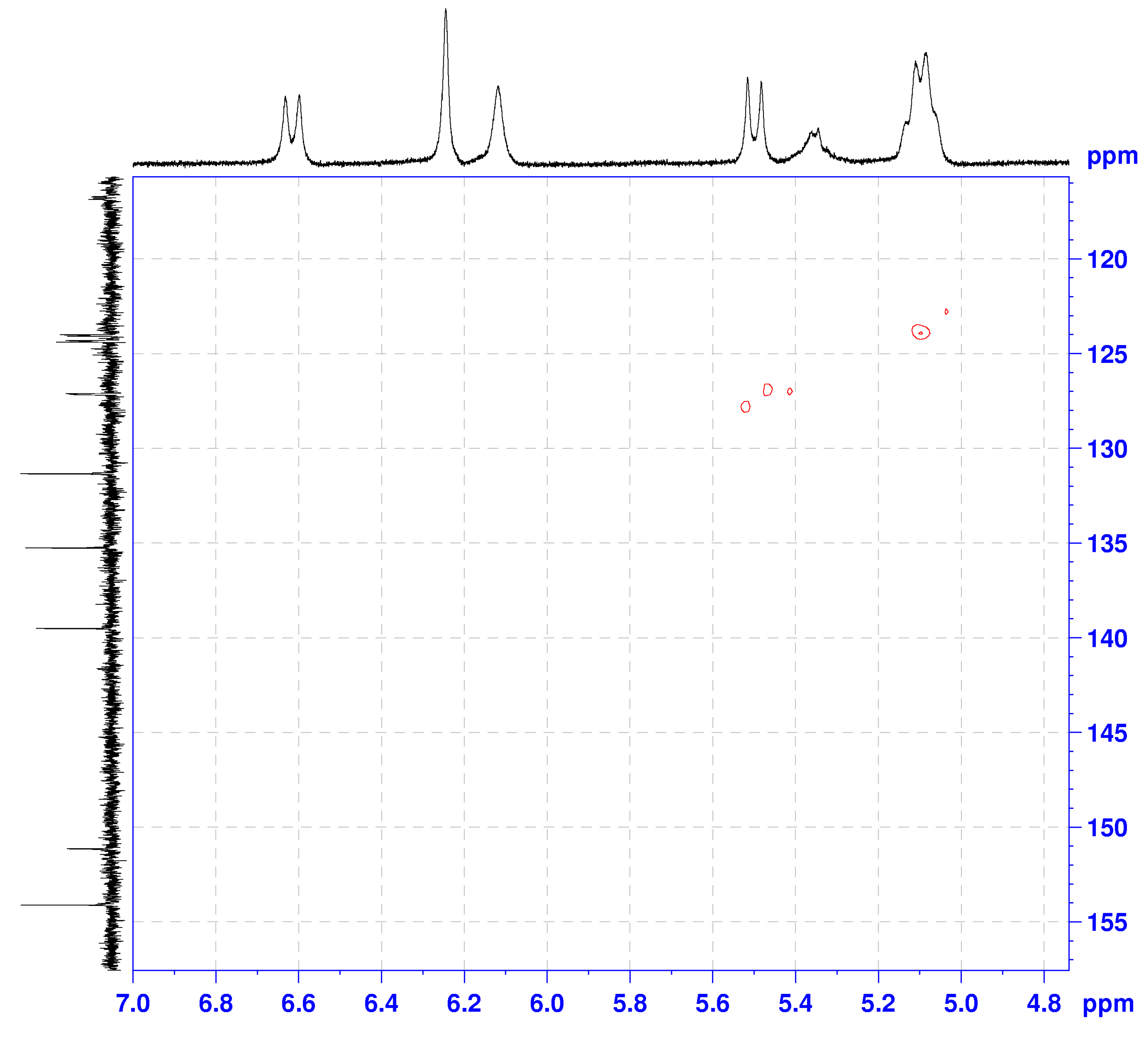

Supplement: S17 Fig — (TIF) [file pone.0231948.s017.tif]

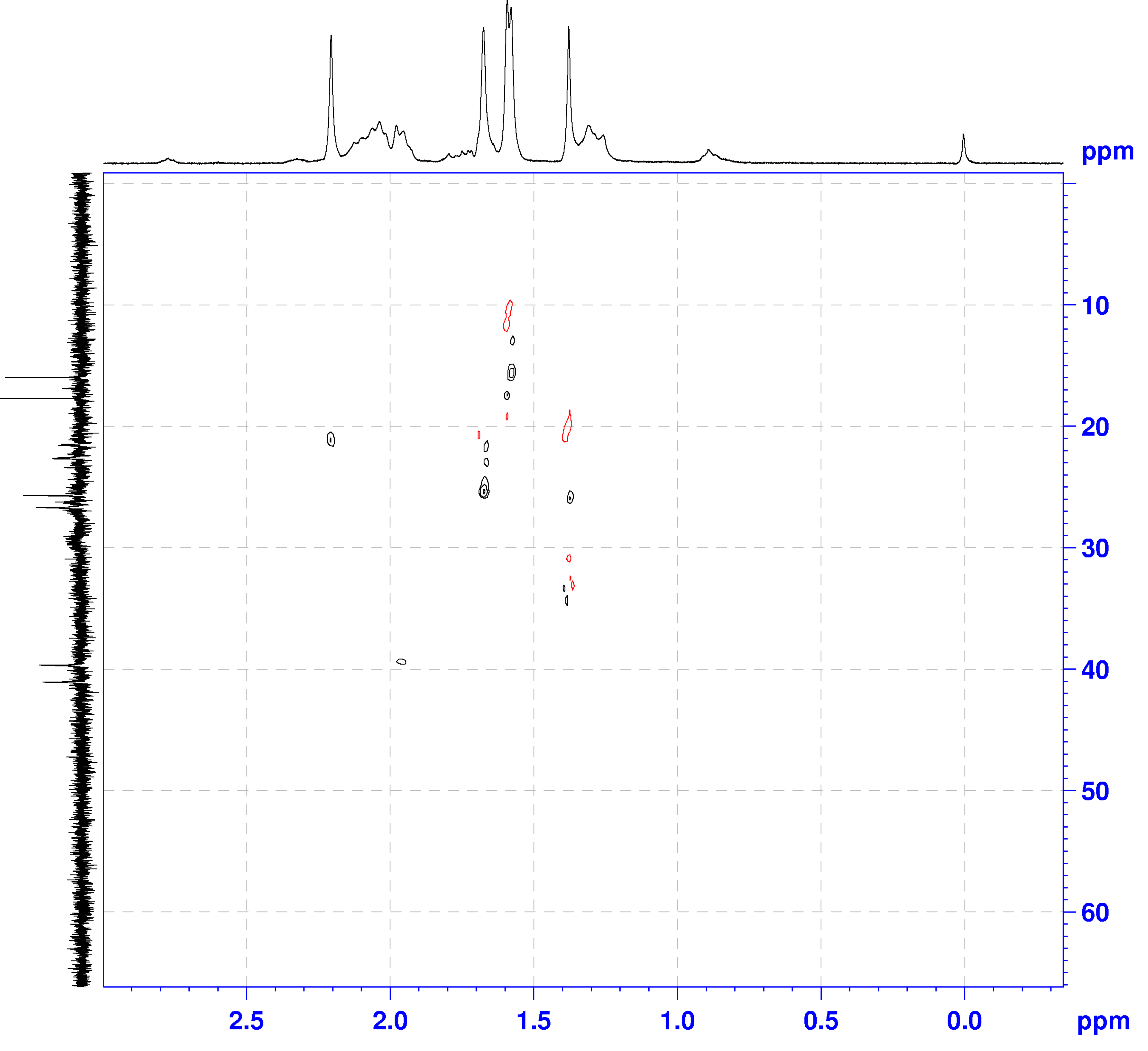

Supplement: S18 Fig — (TIF) [file pone.0231948.s018.tif]

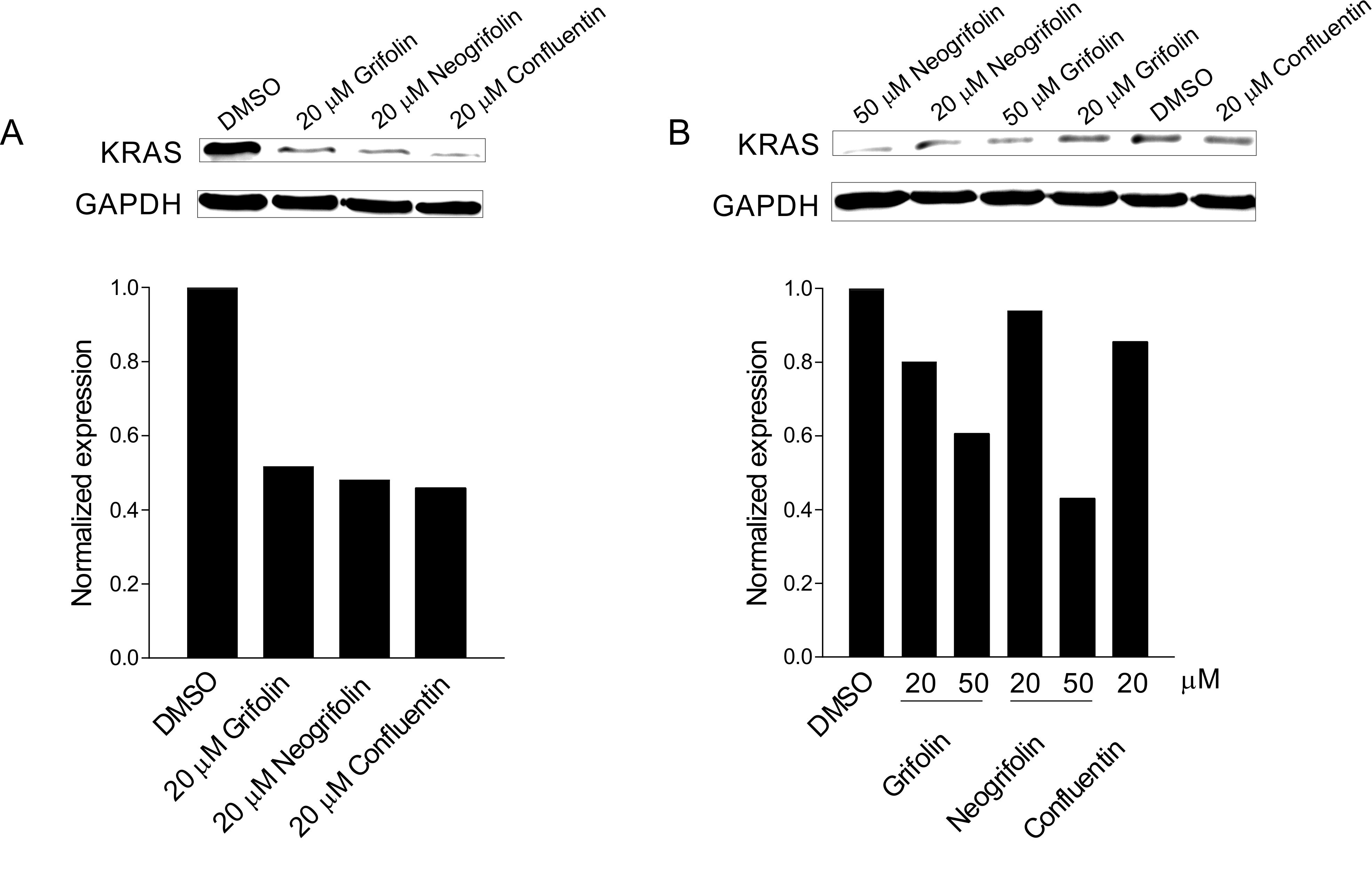

Supplement: S19 Fig — HT29 cells were treated with 20 μM (A) or 50 μM (A and B) of grifolin, neogrifolin and confluentin for 48 hours. Isolated cell lysates were then subjected to Western blot analysis. (TIF) [file pone.0231948.s019.tif]

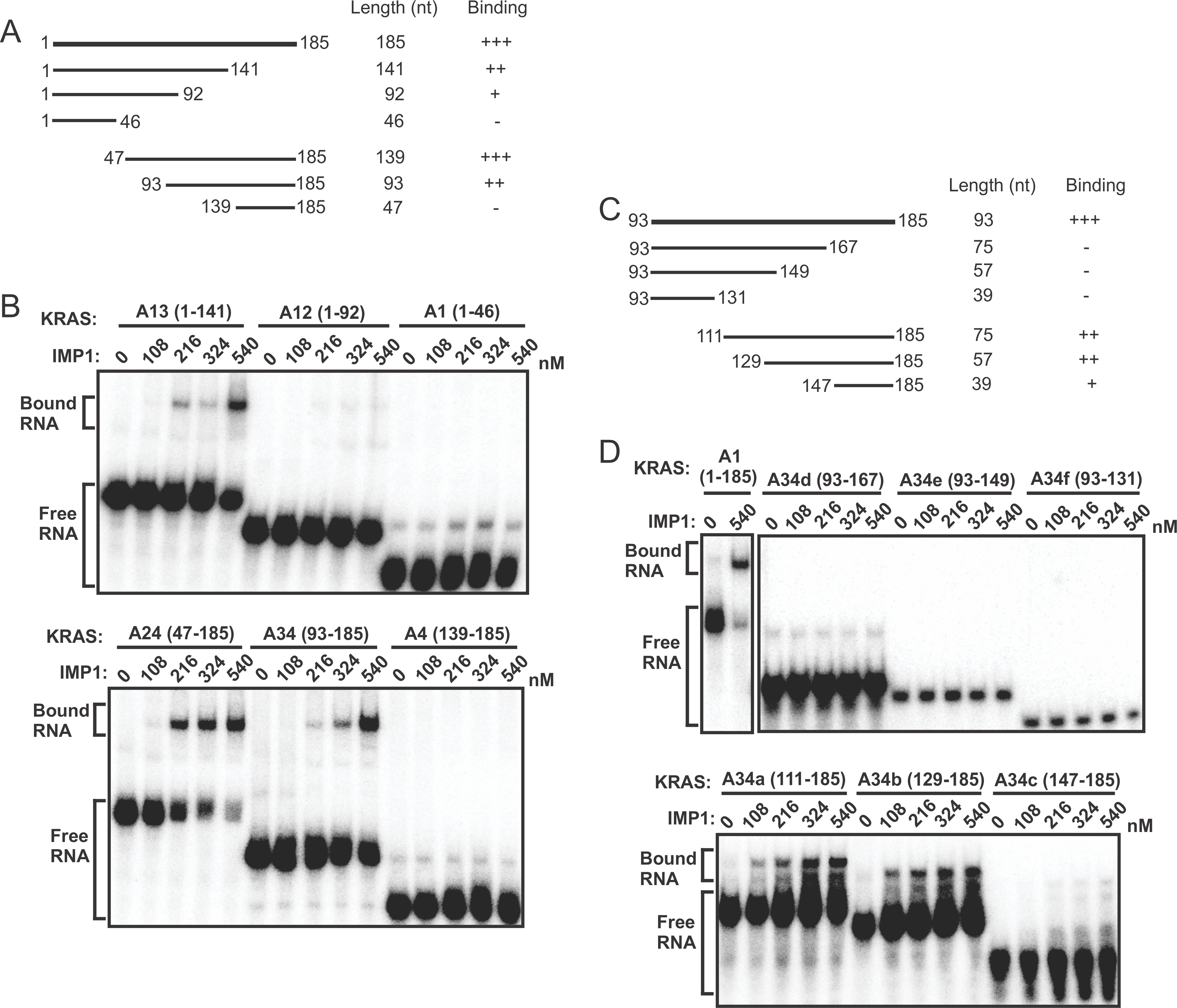

Supplement: S20 Fig — (A) A summary of 3’ and 5’ end truncated KRAS RNA fragments corresponding to nts 1–185, and their relative binding affinity for IMP1. (B) Electrophoretic mobility shift assay of 3’ and 5’ end truncated KRAS RNA fragments corresponding to nts 1–185. (C) A summary of 3’ and 5’ end truncated KRAS RNA fragments corresponding to nts 93–185, and their relative binding affinity for IMP1. (D) Electrophoretic mobility shift assay of 3’ and 5’ end truncated KRAS RNA fragments corresponding to nts 93–185. (TIF) [file pone.0231948.s020.tif]
