# Supplementary material for: Grifolin, neogrifolin and confluentin from the terricolous polypore Albatrellus flettii suppress KRAS expression in human colon cancer cells
Source: PLoS One. 2020 May 5;15(5):e0231948. doi: 10.1371/journal.pone.0231948 (PMC7199964; doi:10.1371/journal.pone.0231948)
Supplement: S1 Table — (DOCX) [file pone.0231948.s021.docx]

**S1 Table.** ^13^C NMR (300 MHz) data of purified grifolin, neogrifolin and confluentin as compared to published data.

|  | Grifolin | | Neogrifolin | | Confluentin | |
| --- | --- | --- | --- | --- | --- | --- |
|  | ẟ_C_ (CDCl3) | | ẟ_C_ (CDCl3) | | ẟ_C_ (CDCl3) | |
| position | This study | Published study^a^ | This study | Published study^b^ | This study | Published study^c^ |
| 1 | 109.08 | 109.3 d | 109.69 | 109.9 d |  |  |
| 2 | 137.57 | 137.2 s | 154.15 | 154.1 s | 78.21 | 78.2 s |
| 3 | 109.08 | 109.3 d | 101.05 | 101.2 s | 127.18 | 127.2 d |
| 4 | 154.81 | 154.6 s | 155.39 | 155.2 s | 116.76 | 116.7 d |
| 4a | - | - | - | - | 106.74 | 106.8 s |
| 5 | 110.37 | 111 s | 117.96 | 118.4 s | 151.02 | 151.0 s |
| 6 | 154.81 | 154.6 s | 138.52 | 138.7 s | 108.4 | 108.3 d |
| 7 | - | - | - | - | 139.55 | 139.5 s |
| 8 | 21.04 | 21 q | 20.19 | 20.2 q | 109.87 | 109.3 d |
| 8a | - | - | - | - | 154.1 | 154.1 s |
| 1’ | 22.18 | 22.3 t | 25.13 | 25.2 t | 41.06 | 41.0 t |
| 2’ | 121.62 | 122 d | 122 | 122.3 d | 22.61 | 22.6 t |
| 3’ | 139.05 | 138.4 s | 137.72 | 137.3 s | 124.06 | 124.0 d |
| 4’ | 39.70 | 39.7 t | 39.68 | 39.8 t | 135.27 | 135.3 s |
| 5’ | 26.67 | 26.7 t | 26.41 | 26.6 t | 39.68 | 39.7 t |
| 6’ | 123.62 | 123.7 d | 123.75 | 123.9 d | 26.68 | 26.7 t |
| 7’ | 135.63 | 135.4 s | 135.47 | 135.4 s | 124.38 | 124.4 d |
| 8’ | 39.70 | 39.7 t | 39.68 | 39.8 t | 131.36 | 131.3 s |
| 9’ | 26.67 | 26.7 t | 26.69 | 26.8 t | 25.7 | 25.7 q |
| 10’ | 124.43 | 124.5 d | 124.42 | 124.5 d | 17.7 | 17.7 q |
| 11’ | 131.33 | 131 s | 131.36 | 131.4 s | 15.97 | 16.0 q |
| 12’ | 25.74 | 25.7 q | 25.74 | 25.8 q | 26.23 | 26.3 q |
| 13’ | 17.72 | 17.7 q | 17.72 | 17.8 q | 21.48 | 21.5 q |
| 14’ | 16.23 | 16.1 q | 16.27 | 16.4 q | - | - |
| 15’ | 16.06 | 16 q | 16.21 | 16.2 q | - | - |

^a^Ishii N, Takahashi A, Kusano G, Nozoe S. Studies on the constituents of *Polyporus dispansus* and *P. confluens*. Chem Pharma Bull. 1988;36: 2918-2924.

^b^Iwata N, Wang N, Yao X, Kitanaka S. Structures and histamine release inhibitory effects of prenylated orcinol derivatives from *Rhododendron dauricium*. J Nat Prod. 2004;67; 1106-1109.

^c^Liu K, Woggon WD. Enantioselective synthesis of daurichromenic acid and confluentin. Eur.J Org Chem. 2010;2010: 1033-1036.
