# Supplementary material for: Grifolin, neogrifolin and confluentin from the terricolous polypore Albatrellus flettii suppress KRAS expression in human colon cancer cells
Source: PLoS One. 2020 May 5;15(5):e0231948. doi: 10.1371/journal.pone.0231948 (PMC7199964; doi:10.1371/journal.pone.0231948)
Supplement: S2 Table — (DOCX) [file pone.0231948.s022.docx]

**S2 Table.** ^1^H NMR (300 MHz) data of purified grifolin, neogrifolin and confluentin as compared to published data.

|  | Grifolin | | Neogrifolin | | Confluentin | |
| --- | --- | --- | --- | --- | --- | --- |
|  | ẟ_H_ (*J* in Hz) | | ẟ_H_ (*J* in Hz) | | ẟ_H_ (*J* in Hz) | |
| position | This study | Published study^a^ | This study | Published study^b^ | This study | Published study^c^ |
| 1 | 6.23 | 6.2 s | 6.26 d (3.0) | 6.23 d (3.0) | - | - |
| 2 | - | **-** | **-** | - | - | - |
| 3 | 6.23 s | 6.20 s | 6.21 d (3.0) | 6.18 d (3.0) | 5.48 d (10) | 5.49 d (10) |
| 4 | **-** | **-** | **-** | - | 6.60 d (10) | 6.60 d (10) |
| 6 | **-** | - | - | - | 6.12 s | 6.11 s |
| 8 | 2.21 s | 2.21 s | 2.23 s | 2.21 s | 6.24 s | 6.24 s |
| 1’ | 3.37 d (7.0) | 3.37 d (7.0) | 3.27 d (7.0) | 3.27 d (7.0) | 1.67–1.78 m | 1.65-1.78 m |
| 2’ | 5.23 t (7.0) | 5.26 t (7.0) | 5.12 t (7.0) | 5.11 t (7.0) | 2.03-2.12 m | 2.01-2.13 m |
| 3’ | - | - | - | - | 5.08 m | 5.06 m |
| 4’ | 1.96-2.12 m | 1.9-2.10 m | 1.84-2.04 m | 1.84-2.04 m | - | - |
| 5’ | 1.96-2.12 m | 1.9-2.10 m | 1.84-2.04 m | 1.84-2.04 m | 1.95 m | 1.95 m |
| 6’ | 5.05 m | 5.06 m | 5.05 m | 5.04 m | 2.03-2.12 m | 2.01-2.13 m |
| 7’ | - | - | - | - | 5.11 m | 5.11 m |
| 8’ | 1.96-2.12 m | 1.9-2.10 m | 1.84-2.04 m | 1.84-2.04 m | - | - |
| 9’ | 1.96-2.12 m | 1.9-2.10 m | 1.84-2.04 m | 1.84-2.04 m | 1.67 s | 1.67 s |
| 10’ | 5.05 m | 5.06 m | 5.05 m | 5.04 m | 1.59 s | 1.59 s |
| 11’ | - | - | - | - | 1.58 s | 1.57 s |
| 12’ | 1.67 s | 1.66 s | 1.67 s | 1.66 s | 1.37 s | 1.37 s |
| 13’ | 1.59 s | 1.57 s | 1.58 s | 1.58 s | 2.20 s | 2.20 s |
| 14’ | 1.58 s | 1.57 s | 1.58 s | 1.58 s | - | - |
| 15’ | 1.81 s | 1.80 s | 1.79 s | 1.80 s | - | - |

^a^Ishii N, Takahashi A, Kusano G, Nozoe S. Studies on the constituents of *Polyporus dispansus* and *P. confluens*. Chem Pharma Bull. 1988;36: 2918-2924.

^b^Iwata N, Wang N, Yao X, Kitanaka S. Structures and histamine release inhibitory effects of prenylated orcinol derivatives from *Rhododendron dauricium*. J Nat Prod. 2004;67; 1106-1109.

^c^Liu K, Woggon WD. Enantioselective synthesis of daurichromenic acid and confluentin. Eur.J Org Chem. 2010;2010: 1033-1036.
