# Supplementary material for: Grifolin, neogrifolin and confluentin from the terricolous polypore Albatrellus flettii suppress KRAS expression in human colon cancer cells
Source: PLoS One. 2020 May 5;15(5):e0231948. doi: 10.1371/journal.pone.0231948 (PMC7199964; doi:10.1371/journal.pone.0231948)
Supplement: S1 Raw Images — (PDF) [file pone.0231948.s023.pdf]

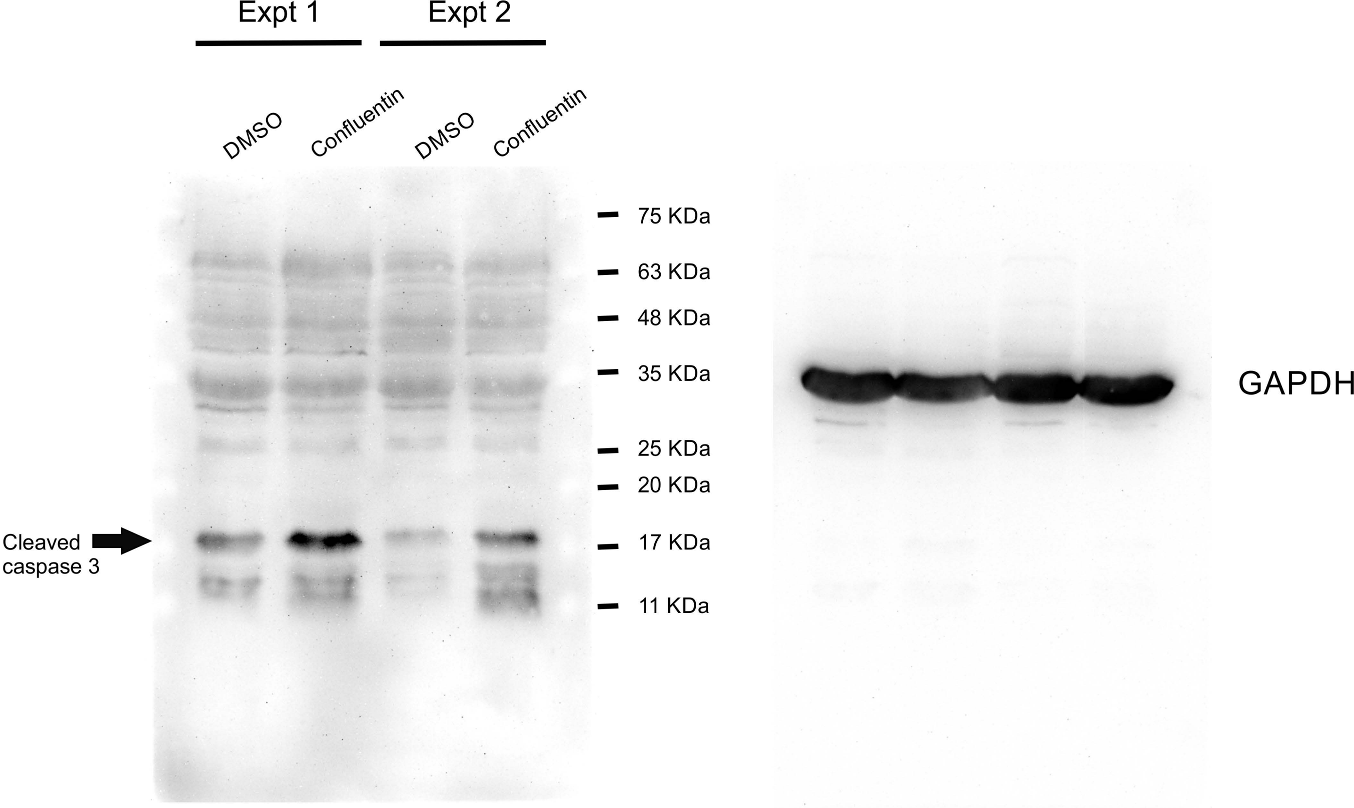

Raw data for Fig 4C. Blot was first incubated with anti-Cleaved Caspase-3 antibody (left image). Then, reprobed with anti-GAPDH antibody (right images). Two biological replicates are shown.

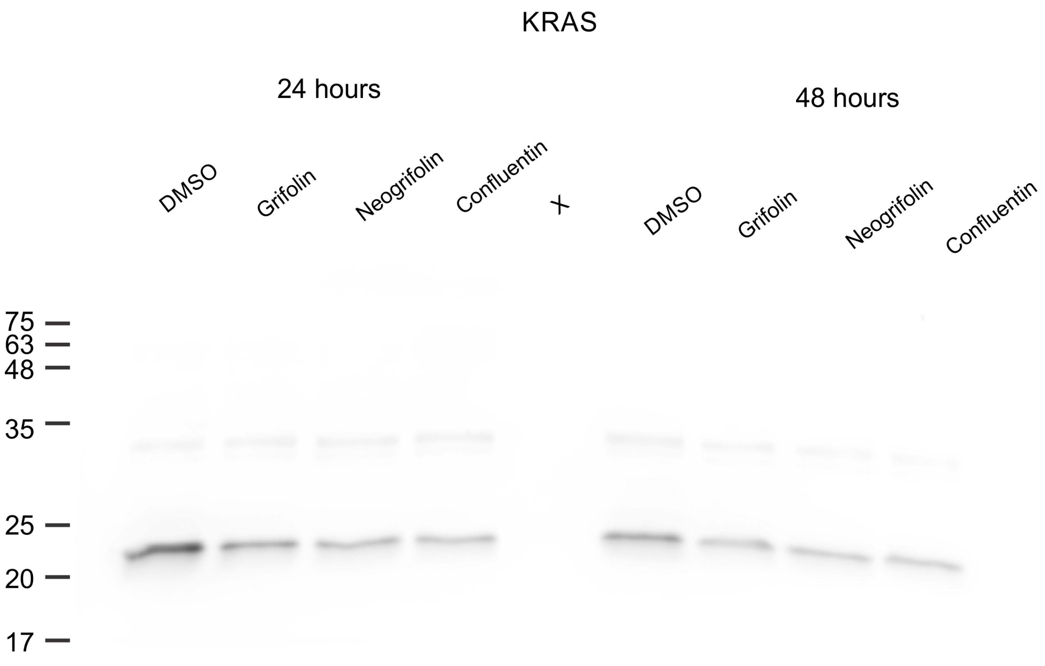

Raw data for Fig 6. Blot was incubated with anti-KRAS antibody

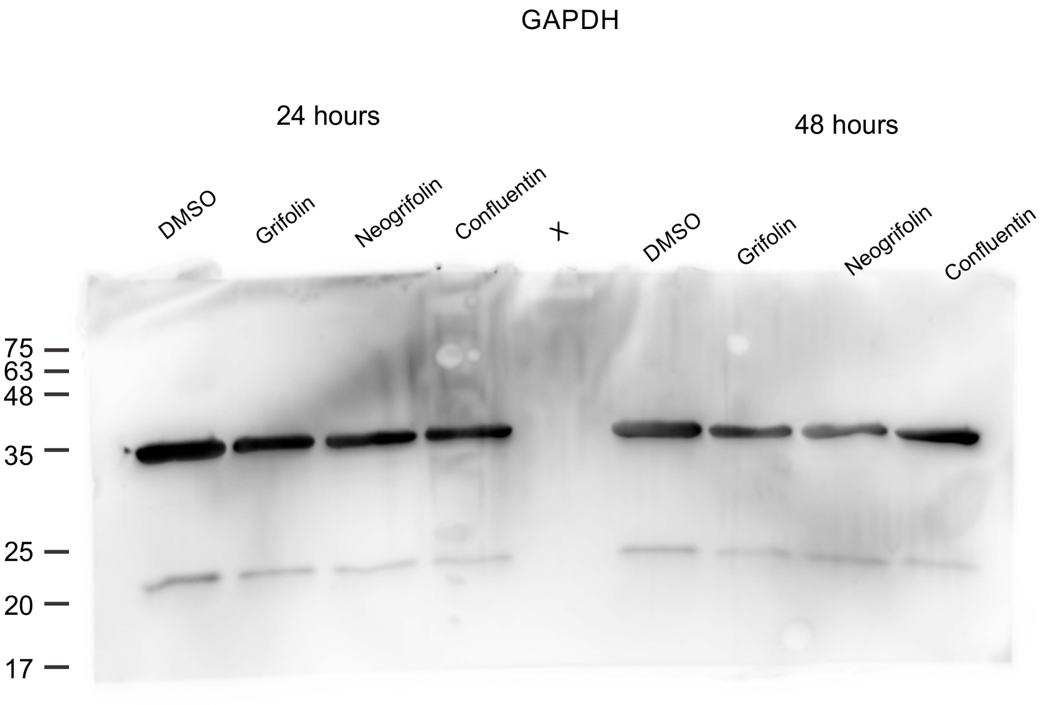

Raw data for Fig 5. Blot was incubated with anti-GAPDH antibody

KRAS

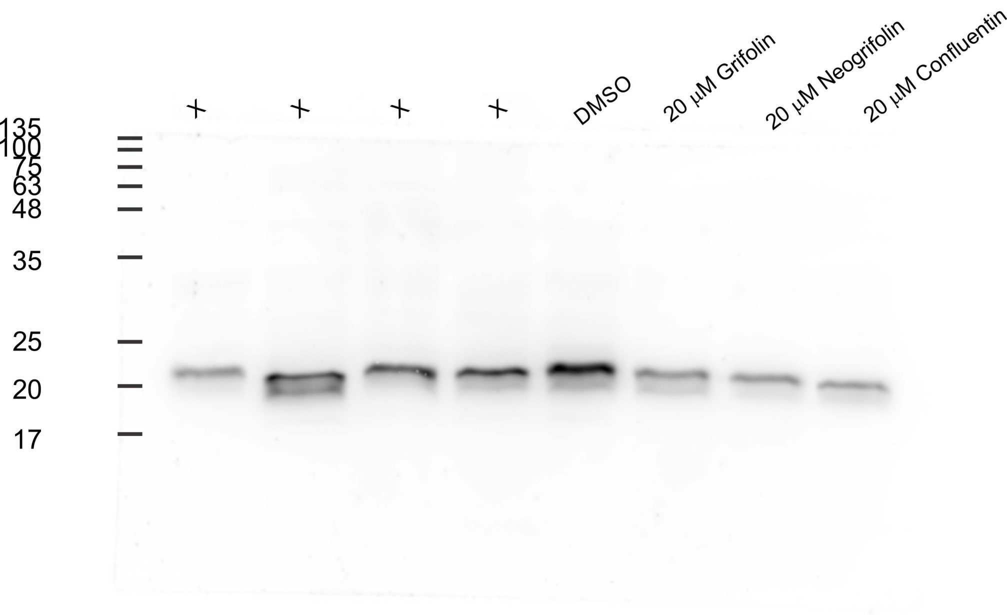

Raw data for S19A Fig. Blot was incubated with anti-KRAS antibody

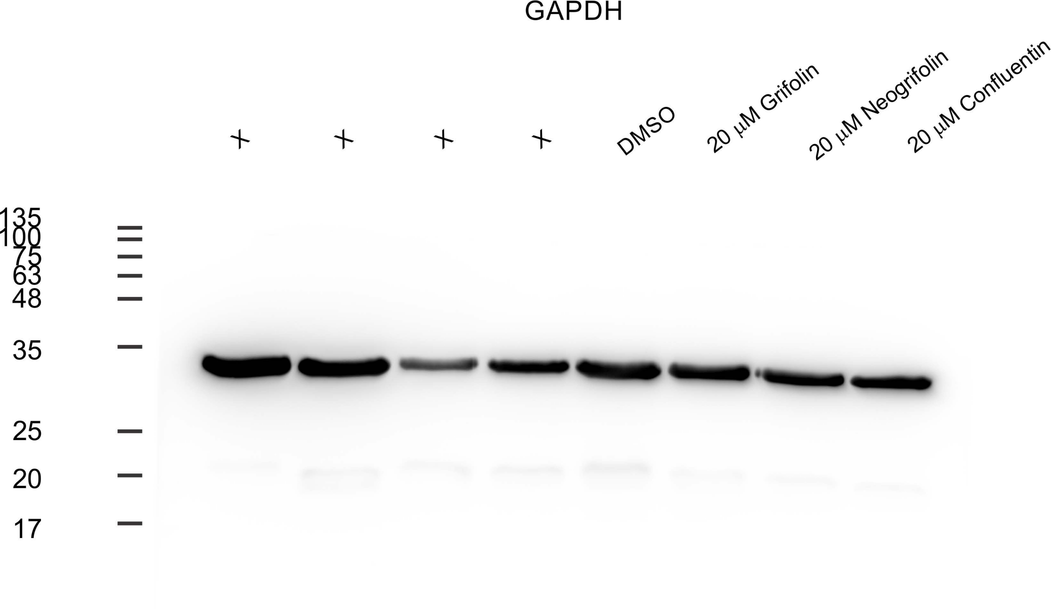

Raw data for S19A Fig. Blot was incubated with anti-GAPDH antibody

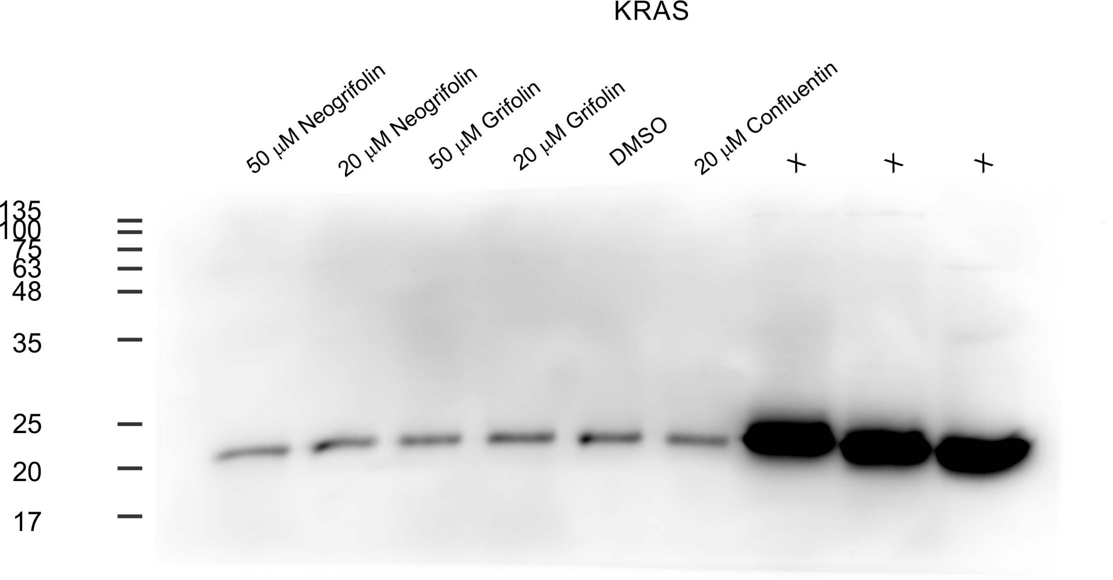

Raw data for S19B Fig. Blot was incubated with anti-KRAS antibody

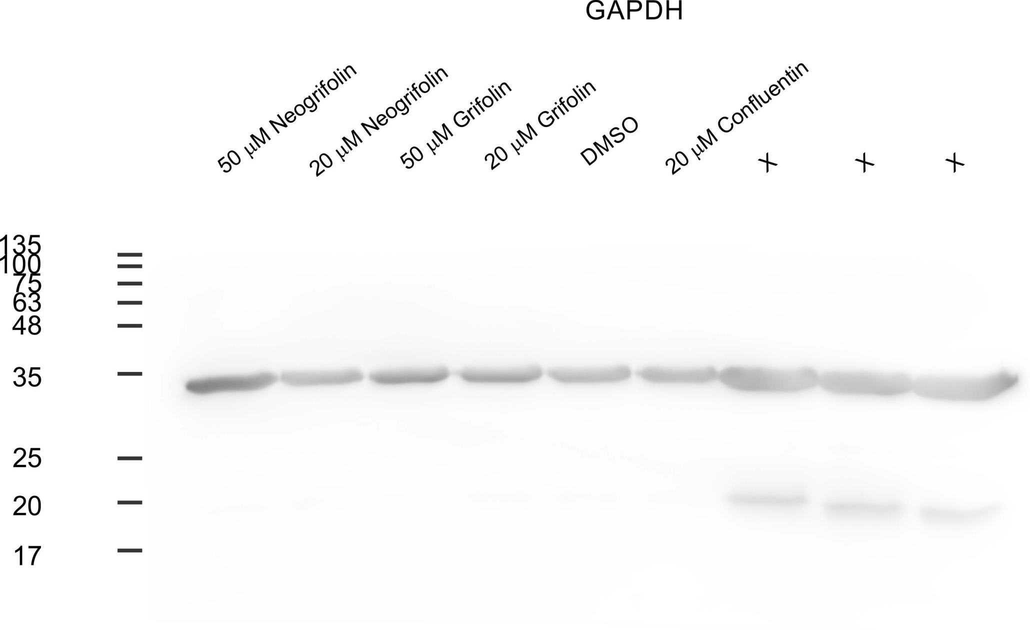

Raw data for S19B Fig. Blot was incubated with anti-GAPDH antibody

Electrophoretic mobility shift assay

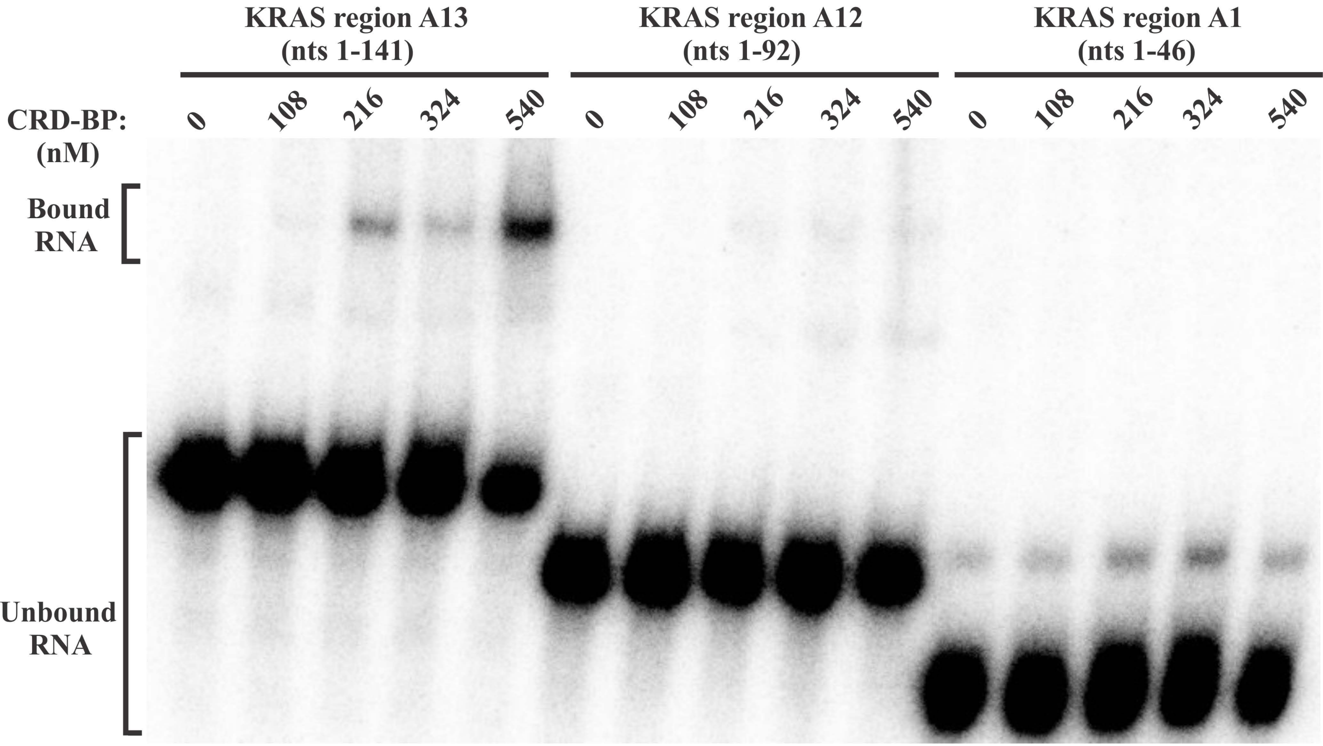

Raw data for S20 B1 Fig.

Electrophoretic mobility shift assay

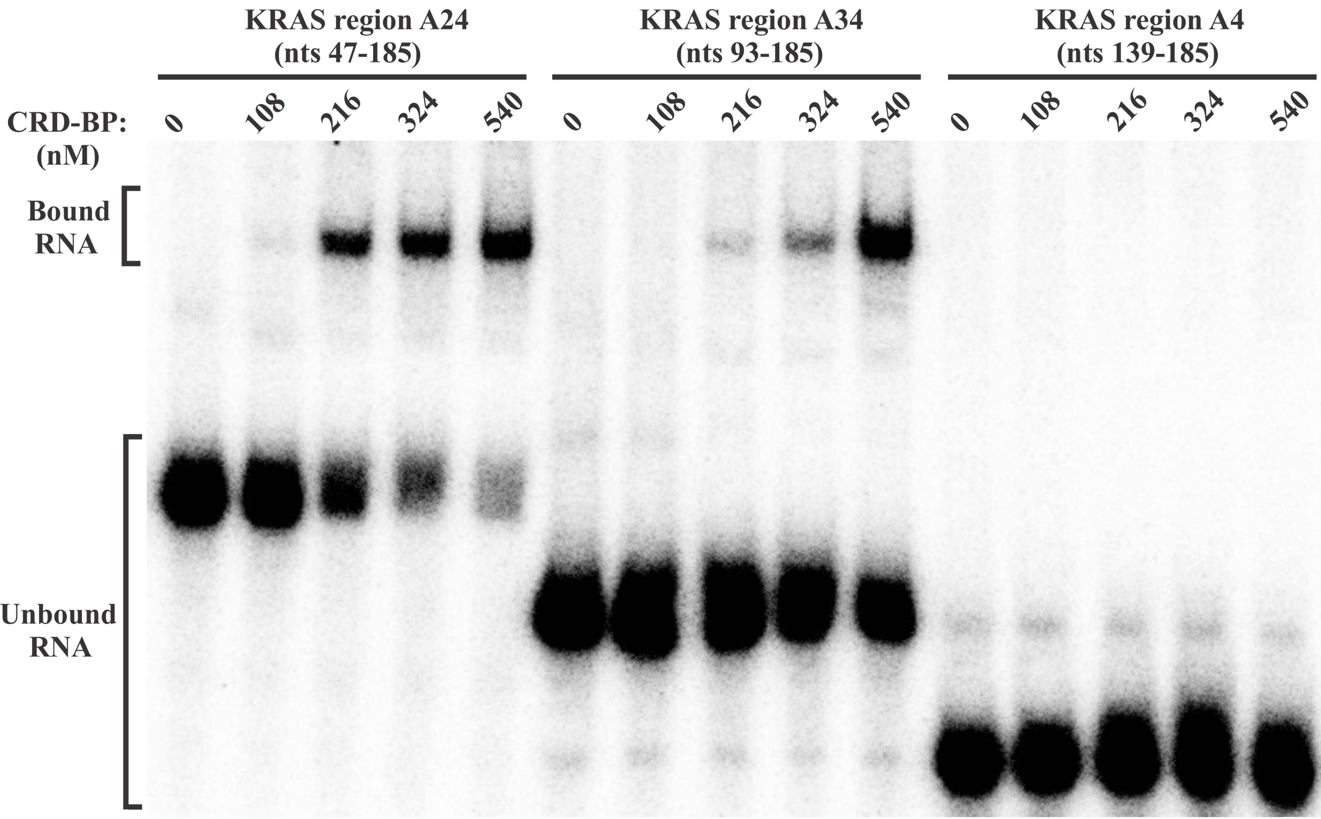

Raw data for S20 B2 Fig.

Electrophoretic mobility shift assay

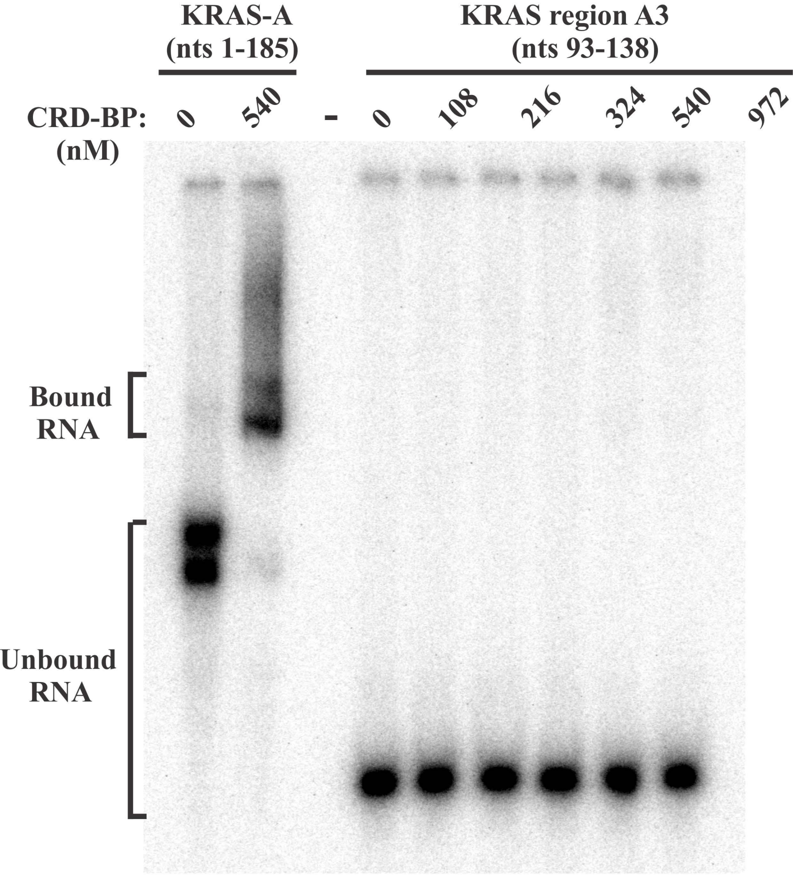

Raw data for S20 B3 Fig.

Electrophoretic mobility shift assay

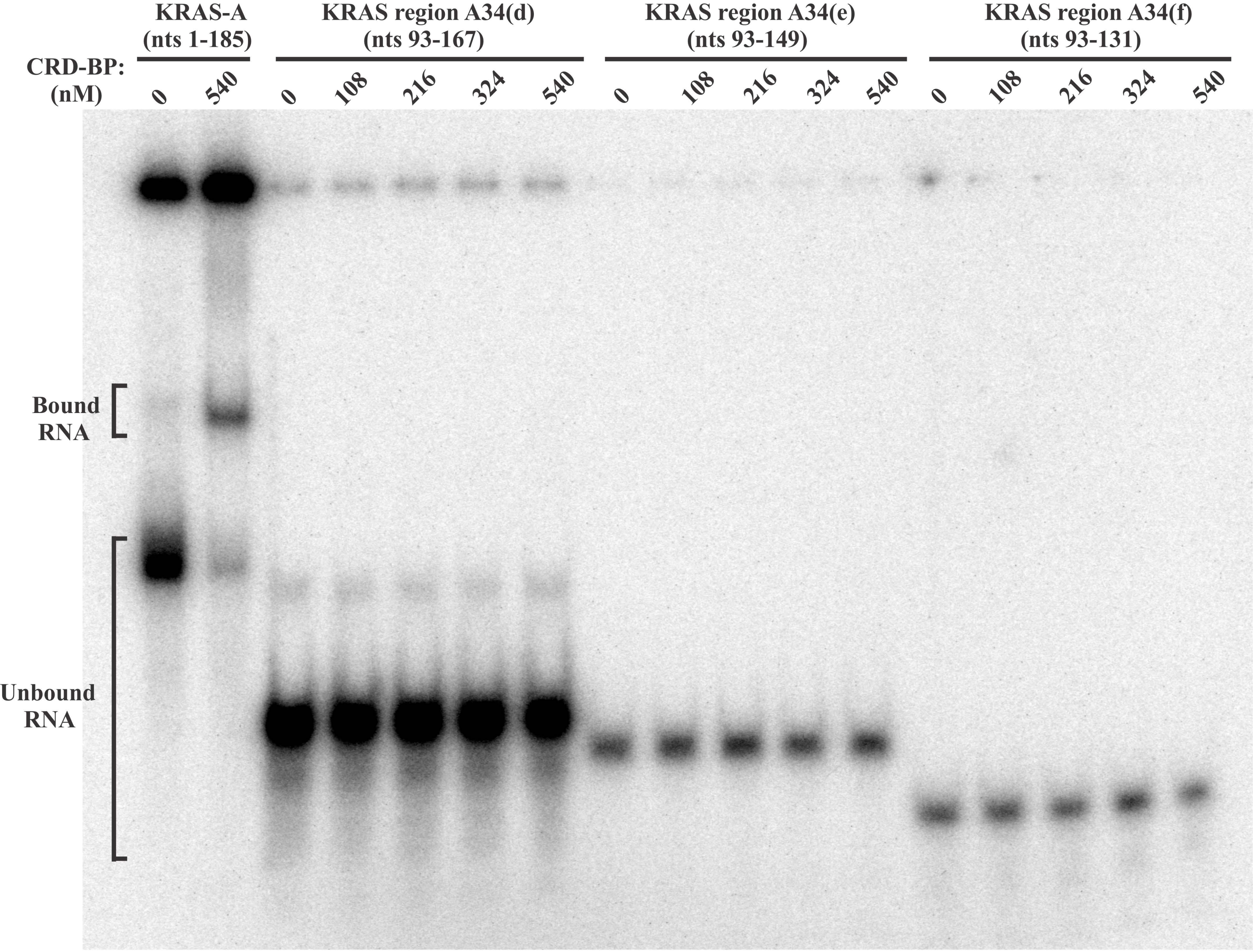

Raw data for S20 D1 Fig.

Electrophoretic mobility shift assay

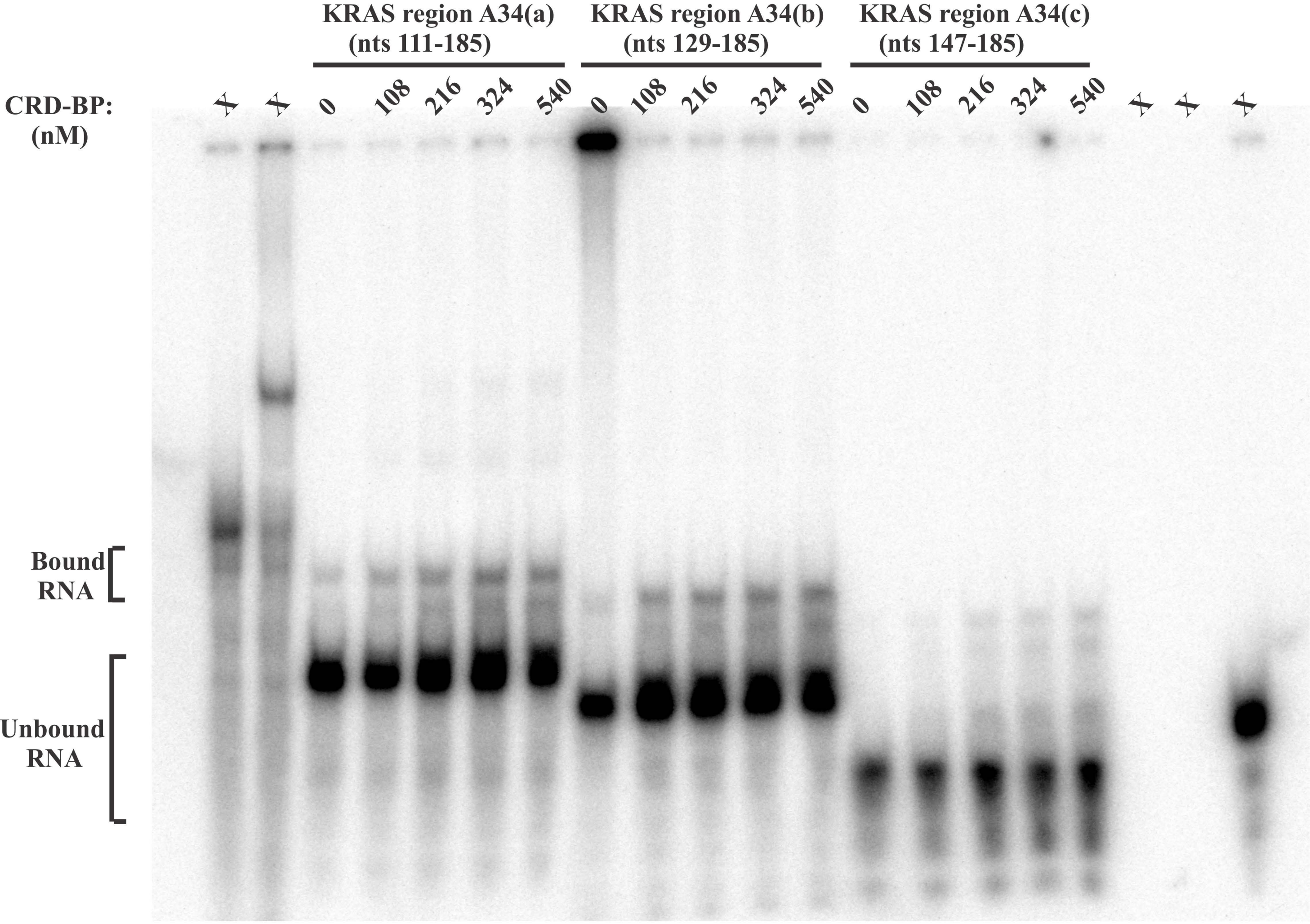

Raw data for S20 D2 Fig
